# Novel Strategy for Structural Variant Genotyping by Short‐Read Genomic Sequencing From Restriction‐Circles: Experimental and Bioinformatics Proof‐of‐Concept

**DOI:** 10.1155/humu/2169350

**Published:** 2026-10-01

**Authors:** Miguel M. Abelleyro, Patricio Yankilevich, Betiana M. Ziegler, Leonela Luce, Héctor M. Cifuentes, Claudia P. Radic, Liliana C. Rossetti, Florencia Giliberto, Carlos D. De Brasi

**Affiliations:** ^1^ Institute of Experimental Medicine (IMEX) Autonomous City of Buenos Aires Argentina; ^2^ Institute for Research in Biomedicine of Buenos Aires (IBIOBA) Autonomous City of Buenos Aires Argentina; ^3^ Institute of Hematological Research (IIHEMA) National Academy of Medicine Autonomous City of Buenos Aires Argentina; ^4^ Faculty of Pharmacy and Biochemistry (FFyB) University of Buenos Aires (UBA) Autonomous City of Buenos Aires Argentina uba.ar; ^5^ Institute of Immunology Genetics and Metabolism (INIGEM) CONICET-University of Buenos Aires (UBA) Autonomous City of Buenos Aires Argentina; ^6^ John Walton Muscular Dystrophy Research Centre Translational and Clinical Research Institute Newcastle University and Newcastle Hospitals NHS Foundation Trust Newcastle upon Tyne UK; ^7^ Patagonian Hospital Translational Knowledge Unit El Calafate High Complexity Hospital-SAMIC El Calafate Santa Cruz Argentina

## Abstract

Structural variants (SVs) comprise copy‐number variants (e.g., deletions and duplications) and copy‐neutral SV (e.g., perfect inversions). Short‐read high‐throughput DNA‐sequencing is the established technology for small‐variant calling in clinical genomics, whereas Oxford nanopore or PacBio long‐read NGS technologies improve SV identification but remain challenging to implement in clinical settings. We developed and validated a novel experimental protocol and bioinformatics pipeline for SV genotyping from short‐read whole‐genome sequencing (WGS) of restriction‐circles of DNA. The experimental protocol includes *Bcl*I‐restriction digestion of genomic DNA, fragment‐ends ligation and short‐read WGS. The bioinformatics pipeline takes advantage of linking pair‐end reads (LPERs), a key operator associating terminal sequences from each restriction fragment. LPERs contain long‐distance haplotype information, essential for SV‐genotyping. Each SV associates with a specific LPER pattern. The approach was applied to a patient with Duchenne muscular dystrophy (DMD) generating proper LPER vertical (8.6x) and horizontal (83%) coverages with good quality standards. SV‐specific LPER quality filters were designed to improve the consistency of SV detection. The approach was validated by verifying a previously characterised *DMD*‐genotype in the proband (*DMD*_exons_45‐54 deletion and *DMD*_exons_38‐43 duplication) with our output. Conventional SV genotyping using other SV detection algorithms can still be successfully applied to complete an accurate definition of the rearrangement. Our SV‐genotyping algorithm detected two novel SVs in Xq28, which were verified by direct molecular investigation, and resulted polymorphic in the general population: a 1.4 kb deletion, allelic frequency AF (CI95%) of 0.83 (0.71–0.91) and a perfect inversion with 0.31 (0.08–0.54) originated by recombination of 99% homologous 8.4 kb inverted repeats (IRs). Although IR‐mediated inversions represent a major challenge for WGS‐based genotyping, our approach correctly indicated the absence of haemophilia recurrent inversions. Additionally, our protocol allows application of regular small‐variant calling. A likely benign SNV in *F8*‐intron‐3 was also identified and verified. The potential clinical utility of this approach is supported by its consistency and ability to improve SV genotyping whilst allowing the application of conventional variant calling algorithms.

## 1. Introduction

Structural variants (SVs) are large rearrangements that may be involved in genome evolution and human diseases. SVs are classified as copy number variants (CNVs), characterized by gain (duplications) or loss of genetic material (deletions); copy‐neutral rearrangements not involving gain or loss of DNA (e.g., perfect inversions, reciprocal translocations); and complex rearrangements, combining characteristics [1–3]. The human genome (HG) presents a vast complexity featured by a high number of long and particularly short interspersed repeated elements (LINEs and SINEs, respectively) and low‐copy number repeats, which are typically present in 2–4 copies per haploid genome [4, 5]. Homologous pairing and recombination between nonallelic copies of these repeats enhance the occurrence of different SVs. SVs mediated by unequal homologous recombination are characterized by being delimited by the repeats and, consequently, the specific breakpoint cannot be precisely defined but remains aligned, allowing a reciprocal exchange [6, 7]. Inversions tend to be flanked by large inverted segmental‐duplications (inverted repeats [IRs]) providing the structural platform to originate perfect, copy‐neutral, inversions (perfect inversions) via nonallelic homologous recombination (NAHR), which are extremely difficult to detect in practice [8]. A paradigmatic example of such perfect inversions is the *F8*‐intron 22 inversion (Inv22), the most prevalent cause of haemophilia, whose diagnosis requires specific approaches for its identification [9, 10].

Next‐generation sequencing (NGS) has revolutionised modern human genetics, so called genomic medicine, by means of performing an accurate and comprehensive genotyping of small variants, including single nucleotide substitutions and indels (SNVs) [11]. However, SV genotyping using NGS, particularly short‐read or second‐generation sequencing platforms, such as Illumina pair‐end read (PER) technology, is still far from reaching its potential, partially due to the prevalent involvement of repeated sequences in the HG [12, 13].

Third‐generation NGS has come to address the challenges posed for SV genotyping by producing much longer reads [14, 15]. However, long‐read or third‐generation sequencing presents practical limitations for its successful application in clinical settings: higher costs, limited global distribution and lower reliability than short‐read NGS for SNV genotyping, and consequently, its insertion in genomic medicine worldwide is still incipient [16, 17].

Notably, for different reasons the challenging haemophilia‐causative Inv22 still remains invisible for second‐ and even for third‐generation NGS. Inspired by the design of inverse shifting‐PCR approach for detection of the Inv22 [18], we developed a new strategy for a comprehensive detection of SVs on a massive scale.

The objective of this work is to present the proof‐of‐concept of a novel approach for SV genotyping by applying short‐read whole‐genome sequencing (WGS) from circularised restriction‐fragment DNA and developing a bioinformatics pipeline for sequence data analysis.

This method constitutes a cost‐effective alternative to long‐read NGS technologies to obtain physical map information and improve SV genotyping.

## 2. Materials and Methods

### 2.1. Ethics Statement

The project was approved by our Institutional Ethics Committee (CEIANM, 13/6/2012). Samples were collected with written informed consent.

### 2.2. Sample Preparation

Genomic DNA (gDNA), from a patient with Duchenne muscular dystrophy (DMD) (control proband) previously genotyped with a deletion of *DMD*‐exons 45–54 and a tandem‐duplication, *DMD*‐exons 38‐43 [19], was extracted from peripheral blood leukocytes (PBL) using CTAB [20]. PBL‐gDNA samples from the general Argentinean population were extracted by the salting‐out method [21]. Purity, concentration and quality of gDNA were assessed by UV‐spectrophotometry (DeNovix DS‐11FX) and agarose gel electrophoresis.

### 2.3. Wet‐Lab Experimental Procedures

A total of 1000 ng of gDNA were digested with 10 U of FastDigest *Bcl*I RE (T′GATCA) (Thermo‐Scientific, Argentina) in 30 *μ*L for 3 h at 37°C, adding a pulse of 10 U of *Bcl*I for 90 min (complete digestion), followed by 20 min at 80°C (heat inactivation). Fragment‐ends′ ligation was performed with 3 Weiss U/0.5 *μ*g gDNA of T4 DNA‐Ligase (Thermo‐Scientific, Argentina) in 200 *μ*L (5 ng gDNA/*μ*L) at 4°C overnight, followed by ethanol precipitation and centrifugation at 4°C for 20 min at 13000 rpm. The precipitated gDNA, *Bcl*I‐circles, was dissolved in 10–15 *μ*L of bidistilled water (90 ng/*μ*L) [22].

WGS was performed with 2 *μ*g of *BclI-*circles on a NovaSeq 6000 and the TruSeq Nano DNA (350) Library (Illumina, San Diego, United States) by Macrogen Genomics (South Korea). A schematic of the experimental workflow is shown in Figure 1A.

**Figure 1:**
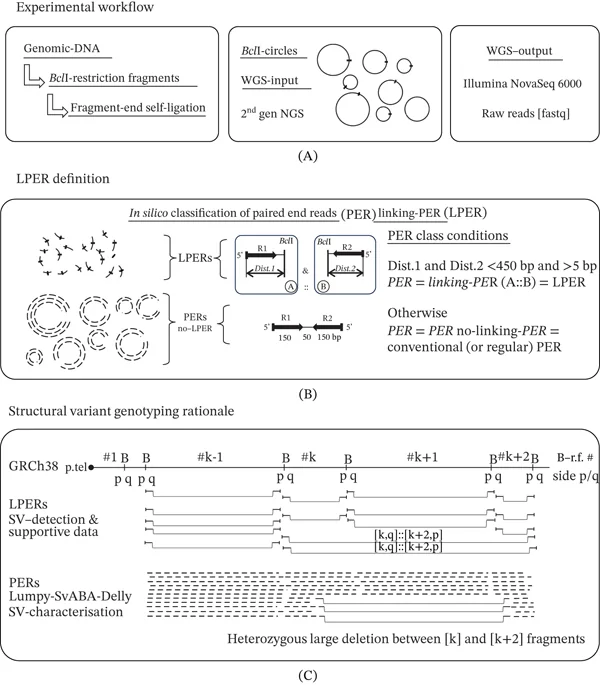
Key procedures for structural variant (SV) genotyping by whole‐genome sequencing (WGS) from restriction circles. (A) wet lab experimental workflow. The first box summarises the obtention of *Bcl*I‐circles from genomic DNA. The second box represents the input for short‐read WGS (2nd gen Illumina platform), and the third box illustrates the output of WGS from the *Bcl*I‐circles (i.e., fastq files with raw reads and their quality attributes). (B) In silico classification of pair‐end reads (PERs). After read mapping to the reference genome, a linking‐PER (LPER) is defined as a PER that links both restriction‐fragment ends in each *Bcl*I‐circle by holding two conditions: the distances from the 5 ^′^‐end of R1 (A part) and R2 (B part) to the proximal *Bcl*I‐restriction sites (Dist. 1 and Dist. 2) are both between 5 and 450 bp. Any other PER are classified as regular/non‐LPER PER, or simply PER. (C) SV genotyping rationale using LPER and PER datasets. B‐r.f.# represents *Bcl*I‐restriction fragment numbered from p‐to q‐telomere (1…N). GRCh38 is the human genome reference. Each LPER connects two *Bcl*I‐restriction fragment terminals [A]::[B]. The schematic shows an example of LPER and PER datasets consistent with a heterozygous large deletion: 2 LPERs connecting fragment# ‘k’ side ‘q’ with fragment# ‘k+2’ side ‘p’. In addition, normal PER dataset complements LPER evidence by application of Lumpy, SvABA and Delly, conventional SV genotyping algorithms that detect three PERs spanning the deletion breakpoint sequence and differences in read coverage in the deleted sequence interval.

### 2.4. SV‐Genotyping Rationale From Short‐Read WGS on Restriction‐Circles

The strategy to improve SV genotyping takes advantage of the juxtaposition of the sequences from both ends of a restriction‐fragment when forming a DNA‐circle. The joining of the ends of each restriction fragment in a circle constitutes a chimeric sequence that represents a long‐distance haplotype. This chimeric sequence, which bridges the two joined restriction sites of a fragment collected in a paired‐end read (PER), constitutes a bipartite operator called linking‐PER or LPER, whose Part [A] linked to Part [B] (Figure 1B and Figure S1). The physical conditions for a PER to set up an LPER [A]::[B] should be that the reads [R1] and [R2] of a PER are both oriented towards the restriction site within 450 bp (Figure 1B and Figure S1). Each LPER part (i.e., A and B) specifies the observed restriction fragment in the sample, according to its order number on the chromosome (from p‐tel to q‐tel, 1…N) and the chromosomal side of the restriction site (i.e., ‘p’ or ‘q’ side). For example, LPERs that join different sides (e.g., p::q) of the same restriction fragment (e.g., k::k) correspond to the reference genomic structure (REF). LPERs that join A::B, where the restriction fragment of A is smaller than B (e.g., k::k+2) and side A is q and B is p, LPER denoted [k,q]::[k +2,p], correspond to a deletion (DEL) involving k and k +2 fragments (e.g., Figure 1C). The assignment of LPERs to the three main types of SVs (i.e., DEL, DUP, INV) is summarised in Table 1. The logic of these LPER/SV assignments is explained in the diagrams of the Figure S2.

**Table 1:** Mapping of linking‐PER (LPER) data supporting the commonest structural variants (SV) and the rationale for the development of the HGVS nomenclature.

Genomic structure	Specific LPER requirement [A]::[B]		HGVS nomenclature ^∗^
REF	[k,p]::[k,q]	=	
DEL	[k,q]::[k+x,p]	=	g.(kqc_kpc)_(k+xqc_k+xpc)del
DUP	[k,p]::[k+x,q]	=	g.(kqc_kpc)_(k+xqc_k+xpc)dup
INV	[k,q]::[k+x,q]	[k,p]::[k+x,p]	g.(kqc_kpc)_(k+xqc_k+xpc)inv

*Note:* ‘kqc’ indicates the genomic coordinate ‘c’ of the restriction site located on the ‘q’ side of the ‘k‐th’ restriction fragment, and ‘k+xpc’, on side ‘p’ of ‘(k+x)‐th’ fragment.

Abbreviations: DEL, deletion; DUP, duplication in tandem; INV, inversion; REF, reference or normal structure.

∗Partially defined SV nomenclature based on restriction fragment intervals of the human genome reference sequence.

### 2.5. Bioinformatics

The bioinformatic analysis is described in detail in the Text S1 and depicted on the flowchart shown in Figure 2. The pipeline included three main processes: data preprocessing, LPER definition and variant calling.

**Figure 2:**
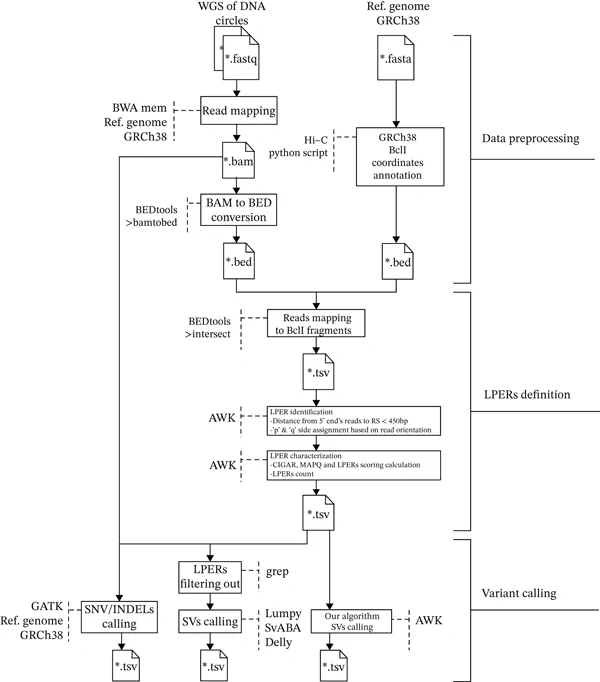
Bioinformatic pipeline to detect structural variants by whole‐genome sequencing (WGS) from restriction circles. Downward workflow including three major processes: Data preprocessing, LPER definition and variant calling. WGS output are two  ^∗^.fastq files (raw reads associated with experimental base qualities) and the human reference genome (GRCh38,  ^∗^.fasta file). BAM acronym represents binary alignment map; BED, browser extensible data; tab separated value ( ^∗^.tsv) file compiles the information of LPERs (linking‐PER) containing relevant attributes. Each LPER is associated with a specific SV hypothesis (e.g., DEL, DUP and INV) according to Table 1′s rules. LPER related information, SV hypothesis assigned, SV call reliability parameters (e.g., LPER counts per SV) and the resulting SVs are compiled in a aain LPER table. Characterisation of all type of variants is completed using conventional variant calling algorithms on regular PERs using GATK (Genome Analysis Toolkit) for SNV/indels and Lumpy, SvABA and Delly for complementing and refining SV calling. A detailed description of the variant genotyping pipeline applying short‐read WGS on restriction circles is shown in the Text S1.

The data preprocessing step starts with two types of input files: fastq files with the output data from applying short‐read WGS to DNA restriction circles and a fasta file with data from the HG reference GRCh38. Subsequently, this step includes mapping the reads to the GRCh38 and obtaining the BclI‐sites from the reference genome.

The LPER definition process takes advantage of the BEDtools intersect command to merge the relative information of WGS reads and *Bcl*I‐site locations on each chromosome. Measuring the distances (Dist.1 and Dist.2) between the 5 ^′^ end of each read (R1 and R2) and the nearest restriction site in the corresponding orientation using AWK programming language, allows us to classify which PER is an LPER and which are not (i.e., Dist.1 and Dist.2 < 450 bp, Figure 1B and Figure S1).

The variant calling process focuses on the characterisation of SNVs/indels using the tools recommended by GATK, as well as the detection/characterisation of SVs through conventional SV‐genotyping programmes (Lumpy, SvABA and Delly) combined with the long‐range haplotype evidence provided by LPER operators (Figure 1C).

A detailed specification of the bioinformatic procedures and the general Linux Bash script for variant detection and characterisation is shown in Text S1 and Text S2, respectively.

### 2.6. Experimental Efficiencies and LPER Coverage

The efficiency of the procedures was assessed by the relative count of X‐linked Normal/reference *Bcl*I‐LPERs versus LPERs counted from the uninvolved *Nco*I (C ^′^CATGG) by applying the same algorithm. Normalisation was achieved using *Bcl*I/*Nco*I ratio of restriction sites in the X chromosome, 1.04 (36,390/35,081). These estimations were done using LPER tables and Microsoft Excel Spreadsheet editor. Vertical‐ and horizontal‐LPER coverages, and Manhattan plots were evaluated using R‐packages (ggplot and qqman).

## 3. Results

The new approach for SV genotyping (Figure 1C) was applied to a hemizygous patient with DMD. The output resulted in 24 comprehensive LPER chromosome tables [23], which compile all LPER′s attributes. To test the efficacy of the entire strategy, most investigations were focused on the X chromosome: variant predictions, method validation and other investigations, such as the inclusion of already proven software tools for SV and SNV/indel genotyping.

### 3.1. Experimental Process Impact Estimation

The impact of the experimental and bioinformatics procedures was estimated by an in‐house designed metrics on normal‐reference X‐linked LPERs: The relative proportion of *Bcl*I‐LPERs (observed/expected) versus the same proportion of *Nco*I‐LPERs, an uninvolved RE. After *Bcl*I/*Nco*I ratio normalisation, our X‐linked‐based metrics allowed estimation of 2.54 templates‐per‐circle or about 40% of bona fide LPERs per fragment copy.

### 3.2. LPER Coverage Analysis

Due to fragment length impacts circularization efficiency and LPER coverage, a series of genome‐wide Normal/reference *Bcl*I‐LPER estimations were assessed. Figure 3A shows the vertical LPER coverage (depth), (total 11,617,479/766,417, average 8.6x LPERs/fragment). This vertical coverage estimation of LPERs shapes a Poisson‐like distribution that distinguishes a region of maximal coverage, *Bcl*I‐fragments between 0.5–17 kb associated with an average depth of 11.8x, and two regions of minimal coverage, < 0.5 and > 17 kb, with a mixed average of 2.5x.

**Figure 3:**
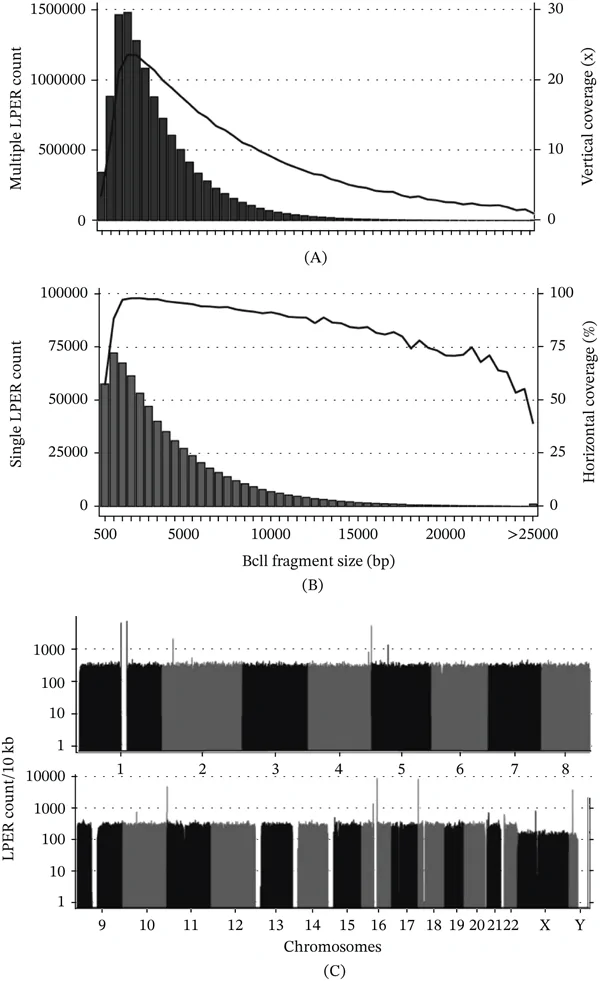
LPER coverage per *Bcl*I fragment length on the reference human genome. (A) Histogram of LPER vertical coverage: multiple LPER count per fragment (grey bars) and LPER depth per fragment (e.g., the global average of 8.6x) (black line). *Bcl*I fragment/circles length bins were classified into 500 bp‐long intervals. B. Histogram of LPER horizontal coverage: single fragment LPER count (a fragment with 1 or more LPERs adds a single count) (grey bars) and their related percentage to fragment/circles number in GRCh38 (e.g., the global average of 86%) (black line). *Bcl*I fragment/circles length bins were equally classified in intervals. C. Manhattan plot showing the horizontal coverage of normal LPERs on the human genome. The graph shows vertical grey lines representing the Log10 of LPER count per 10 kb‐long intervals through the reference GRCh38.p14: 22 autosomes (Chr 1–Chr 22), the X and the Y chromosomes (3200 Mb). Note that the average LPERs counts from autosomes exceeded those from sex chromosomes, X and Y, as the genomic DNA sample corresponded to a male patient 46, XY.

Figure 3B shows the horizontal LPER coverage (computing fragments with at least one LPER; total 680,960) representing about 83% of *Bcl*I‐fragments in GRCh38. This latter average results from an uneven distribution of LPER coverage: *Bcl*I‐fragments < 0.5 kb averaged 57% and those > 20 kb averaged 67% (range 39%–80%), although fragments range 0.5–20 kb, with 91% of fragments covered (range 88%–99%) (Figure 3B). As expected, the poorest covered LPER‐fragments are those > 25 kb (average coverage 39% and vertical, 0.8x).

Figure 3C shows the LPER horizontal coverage through the reference genome (Manhattan plot). Euchromatic regions typically show an adequate range of normal/reference LPER coverage (averaged 172 LPERs per 10 kb bins); highly repeated regions show an extremely high number of counts on their *p*‐ and *q*‐boundaries of the motif, whereas satellite sequences within the *p*‐arms of acrocentric chromosomes (Chr. 13, 14, 15, 21 and 22) and centromeric regions with highly repeated sequences are characterised by overrepresented boundaries and tend to show an artificially poor or even nil central LPER coverage.

### 3.3. Performance and Validation

The performance of the method applied to the control DMD proband was analysed on the X chromosome, obtaining 294,196 LPERs. Table 2 shows that 91% of raw LPERs support the normal/reference structure (REF). The application of stringent SV type‐specific filters to get bona fide LPERs helped in defining DELs, DUPs and INVs, the most prevalent SVs (Table 2). Read quality filters warrant a consistent mapping and LPER count (> 2) excludes random restriction‐fragment associations. A key feature of the SV genotyping system based on LPER is related to the handling of repetitive DNA sequences, which are promoters of recombination and are particularly abundant in the HG, LINE1 (17%), LINE2 (3%), LTR (8%), SINE/Alu (10%) and MIR (2%), covering approximately 40% of GRCh38 [24]. Table 2 shows that of the total LPERs on the X chromosome, 89% include reads that show high mapping quality and consequently unrelated to repetitive sequences of the genome (i.e., MAPQ > 30). Furthermore, LPERs that represent the REF also show a similar parameter of 92% and those associated with typical SVs (i.e., DEL, DUP and INV), more than 73%. The application of this quality filter illustrates the actual effectiveness of our system in dealing with repetitive sequences (e.g., LINE, LTR, SINE and MIR) to obtain these long‐range haplotypes. The application of the filter |*D* *i* *s* *t*| ≥ 30 *b* *p* selected only those LPERs whose component [A] and [B] parts′ specificities are sufficiently robust. DELs and DUPs involving consecutive fragments exclusion (i.e., k and k+1) avoid assuming two undecidable confounders: RE partial‐digestion representing an experimental artifact or, a restriction fragment length polymorphism (RFLP). An INV specific filter comes from the need to cover at least both required restriction‐fragment breakpoints (p::p and q::q) (Table 1). Once SV‐specific filters are applied, the analysis detects seven X‐linked DELs, five DUPs and three INVs (Table 2).

**Table 2:** Application of filters for obtaining high‐quality LPERs to detect bona fide SVs on the X chromosome.

	X‐linked # LPERs (%)	A and B *M* *A* *P* *Q* ≥ 30 # LPERs (%)	A and B |*D* *i* *s* *t*| ≥ 30 # LPERs (%)	Hypoth. *c* *o* *u* *n* *t* ≥ 3 # LPERs (%)	Special filters # LPERs (%) [# SV]
Total	294,196 (100)	261,737 (89.0)	251,670 (96.2)	237,845 (94.5)	NF
REF	267,830 (91.0)	242,836 (92.8)	240,151 (95.4)	234,143 (99.4)	NF
DEL	3571	2527	2503	721 (20.2)	33 (4.6) [7]
DUP	14,273	11,755	5786	2547 (17.8)	14 (0.5) [5]
INV	2237	381	76	75 (3.4)	18 (24) [3]

Abbreviations: |*D* *i* *s* *t*| ≥ 30, distance from 5 ^′^‐read to the restriction site ≥ 30 bp; DEL, deletion; DUP, duplication; INV, inversion; MAPQ ≥ 30, map quality ≥ 30; NF, no additional filter applied; REF: reference structure; SV, structural variant.

The list of these 15 X‐linked SVs detected in the DMD‐affected patient includes the DEL (*DMD*‐exons 45–54) and DUP (*DMD*‐exons 38–43). Consequently, the standalone utility of the LPER‐based SV genotyping strategy was validated by defining the patient′s DEL as NC_000023.11:g.(31635866_31648675)_(32091845_32095165)del, whose intervals encompass the DEL 5 ^′^‐(31646629) and 3 ^′^‐breakpoint (32093105), and the DUP, g.(32217707_32223942)_(32350672_32355426)dup), spanning the defined 5 ^′^‐(32222433) and 3 ^′^‐coordinates involved (32353717) (Table S1; SV genomic coordinate equivalences, Text S3).

### 3.4. Integration to Conventional SV Genotyping Algorithms

An artificial dataset was generated excluding the LPER′s reads from the original dataset. This new dataset, namely PER‐minus‐LPER, was subjected to conventional SV calling algorithms and complete SV genotyping. Table S2 shows the output of X‐linked detected SVs from the PER‐minus‐LPER dataset as input for Lumpy, SvABA and Delly algorithms [25]. To exercise caution about the results, we filtered‐in only the outputs of the fourth quality percentile Q4 (≥ 75%): Lumpy detected 49/47/28 DEL/DUP/BND (break‐end evidence of undefined SVs); SvABA, 262 BND, and Delly, 95/621/13/9 DEL/DUP/INS/INV (Table S2).

All three algorithms detect the *DMD*‐causative DEL, whereas only Lumpy and Delly detect the DUP (Table S2, corresponding rows are highlighted with green background). The evidence obtained from these specialised SV callers perfectly matches both SV previously characterised [19] and coincides with the evidence of our approach.

### 3.5. Additional X‐Linked SVs

The SV‐genotyping strategy predicted two additional X‐linked SVs with bona fide LPERs NC_000023.11:g.(148420712_148429387)_(148429465_148431193)del and g.(153065931_153075234)_(153075234_153084298)inv (Table S1).

The predicted DEL was further defined by Lumpy–SVaBA–Delly algorithms as g.148428177_148429716del, nickname Xq28/1.5 kb‐del. A specific gap‐PCR assay allowed confirmation of Xq28/1.5 kb‐del in the DMD proband and investigation of 41 individuals (females/males, 17/24) from our general population (Figure S3). Table 3 shows the parameters of Xq28/1.5 kb‐del, which resulted more frequent than the REF and highly polymorphic with an allelic frequency AF(95% CI) of 0.83 (0.71–0.91), showing a nonsignificant excess of heterozygotes (observed/expected, 0.47/0.29, Hardy–Weinberg equilibrium).

**Table 3:** Populational parameters of two SV detected predicted by LPER analysis.

**HGVS SV nomenclature nickname**	**Alt. allele freq. (95CI)**	**Ref. allele freq. (95CI)**	**Obs. Het. (exp. het.)**	***N* (females/males)**	**HW eq.chi sq. test**
Xq28(148Mb)1.5kb‐del					
g.(148420712_148429387)_(148429465_148431193)del (LPER‐based genotype)					
g.148428177_148429716del (Lumpy‐SVaBA‐Delly‐based genotype)					
	0.83 (0.71–0.91)	0.17 (0.09–0.29)	0.47 (0.29)	41 (17/24)	NS *p* > 0.05
Xq28(153Mb)‐inv					
g.(153065931_153075234)_(153075234_153084298)inv (LPER‐based genotype)					
Non detected (Lumpy‐SVaBA‐Delly‐based genotype)					
	0.31 (0.08–0.54)	0.69 (0.46–0.91)	0.6 (0.43)	16 (5/6)	NS *p* > 0.05

*Note:* HGVS, Human Genome Variation Society; SV, structural variant, nomenclated on the reference X‐chromosome sequence, NC_000023.11; Ref./Alt. allele, reference/alternative allele (i.e., indicated SV); (95CI), 95% confidence interval (binomial distribution); HW eq., Hardy–Weinberg equilibrium; N, studied individuals; Obs./Exp. Het., observed/expected heterozygosity (i.e., 2pq).

Remarkably, the predicted INV remains undetected by conventional SV calling algorithms. This lack of evidence from short‐read WGS raised the hypothesis of a perfect inversion, characterised by homologous recombination between IR not associated with chimeric reads or CNVs. Interestingly, the alignment between the involved fragments revealed a large (8.4 kb), highly homologous (99.6%) IR (Figure S4). Consequently, homologous recombination between the centromeric (X:153,066,014–153,074,418) and telomeric (X:153,075,613–153,084,041) IR arms delimitated a 1195 kb inversion, nickname Xq28/1.2 kb‐inv, NC_000024.11:g.(153066014_153074418)_(153075613_153084041)inv.

An IS‐PCR assay confirmed the Xq28/1.2 kb‐inv in the DMD proband sample and allowed investigation of 11 samples from our general population (females/males, 5/6) (Figure S5A–C). Table 3 shows the populational characteristics of Xq28/1.2 kb‐inv: AF(95% CI) of 0.31(0.08–0.54) with an observed/expected heterozygosity of 0.6*/*0.43, in Hardy–Weinberg equilibrium.

### 3.6. Haemophilia Inversion Hotspot Genotyping Prospect

The perfect inversion Inv22—the most prevalent cause of haemophilia—originates from homologous recombination between 10 kb‐long, almost identical (99.9%) repeats named *int22h* (*int22h*‐1, intra‐*F8* copy; *int22h*‐2 and *int22h*‐3, extragenic copies by the Xq‐telomere) [26]. The involvement of *int22h* sequences impedes Inv22 detection by massive high‐throughput analysis. Because the Inv22 can be detected by *Bcl*I‐pattern shifts, our approach was challenged to determine the Inv22 status by analysing the LPERs spanning *int22h*‐1, *int22h*‐2 and *int22h*‐3 (Figure S6). As expected, the DMD proband only showed the normal versions of these LPERs (*int22h*‐1 #40146, ‐2 #40298 and ‐3 #40313) (Table S3 and Figure S6).

Likewise, *F8*‐intron 1 inversion (Inv1) causes 2%–5% of severe Haemophilia As and involves copies of a 1 kb‐long IR. Table S3 shows only LPERs associated with the norma/REF, confirming the absence of the Inv1. These negative results about the Inv22 and Inv1 obtained by analysing the corresponding LPERs were confirmed by specific IS‐PCR assays [27] (Figure S5D).

### 3.7. Integration of SNV/Indel Variant Calling Algorithms

To complete a comprehensive variant genotyping, the original dataset (BAM‐file) obtained from *Bcl*I‐circles was subjected to conventional small variant calling algorithms (SNV/indels). GATK4 germline pipeline indicated several deep‐intronic and other variant types in the *F8* (Table S4). Among these *F8* variants, X:154993157G>A, a nonpathogenic SNP, rs35621875, on the acceptor splicing site of *F8*‐intron 3 (NM_000132.4:c.389‐9C>T), was experimentally confirmed by PCR‐amplification and Sanger sequencing (Figure S7).

## 4. Discussion

We presented the proof‐of‐concept of a novel experimental strategy that extends the capabilities of short‐read NGS technologies for massive SV detection not interfering with conventional variant calling algorithms. The method takes advantage of the long‐distance haplotype information contained in restriction‐circles. It allows the detection and definition of perfect inversions, which are highly prevalent in the HG as they originate from NAHR between IRs and result virtually invisible by NGS‐based genotyping. The practical approach included the development of an ad hoc bioinformatics pipeline to process the long‐distance haplotype information contained in LPERs, PERs linking the ends of each restriction circle, computing extra evidence for SV calling.

Among the best virtues of second‐generation NGS is the accurate and robust genotyping of SNVs. However, Illumina platform short‐read technology, using PERs (targeting tracts of 350–400 bp), counts with only two sources of evidence to detect SVs: (a) read‐depth (vertical‐coverage) and (b) eventual PERs defining SV‐breakpoints, discordant [28] and/or split‐reads [29]. This scarcity of data results insufficient to achieve a proper long‐distance analysis, particularly with the common involvement of repeated sequences with different lengths (0.3–50 kb) in the HG. This material limitation prompted us to add long‐distance haplotype information from another source to short‐read NGS to expand SV detection without compromising SNV genotyping. This latter goal was demonstrated by inputting our set of mapped reads from restriction‐circles to conventional algorithms for SNV‐variant calling, such as those recommended by GATK [30, 31]. Besides, conventional SV genotyping was achieved by subjecting data from a LPER‐extracted BAM file to SV calling using Lumpy‐SVtyper, SvABA and Delly algorithms that help in characterising SV‐sequence scars and eventual CNVs (differential depth) [32–34].

The circularization process, to get restriction‐circles as input for short‐read NGS, represents the key procedure to succeed in obtaining reliable outputs. The literature shows estimations of inverse‐PCR protocol‐based circularisation efficiencies by different metrics. A former paper describing chromosome jumping assessed a circle‐to‐concatemer ratio of 88% [35]. Our group estimated an efficiency range of two to five templates‐per‐circle for particularly defiant *Bcl*I‐fragments (20–21.6 kb) associated with the IS‐PCR Inv22‐genotyping [27].

Our massive X‐linked data estimates a normalised circularisation‐efficiency of 2.54 templates‐per‐circle or 40% to obtain bona fide LPERs per *Bcl*I‐fragment.

The most operative metrics to investigate an NGS‐based procedure come from the analysis of the vertical and horizontal coverages of the genome. The WGS‐Illumina platform offers vertical coverages of 36x for autosomes and 18x for sex‐linked chromosomes. Our genome‐wide metrics allowed estimation of LPER vertical coverage in 8.6x and 83% of horizontal coverage. Manhattan plots of LPERs mainly agree with these estimations. Although LPER coverage estimations appear to be suboptimal, these figures assure a qualitatively–quantitatively adequate long‐range haplotype information for detecting all types of SVs. Moreover, a rearranged restriction fragment with two or more supporting LPERs represents a standalone proof of its actual existence as it cannot be obtained by random fragment association.

The integrated strategy, including conventional variant‐calling algorithms, was validated by providing an accurate characterisation of a *DMD*‐large deletion and a *DMD-*tandem duplication in the patient with DMD, who was previously genotyped by application of several methods, such as DMD‐specific multiplex ligation‐dependent probe amplification (MLPA), genome‐wide SNP‐array, breakpoint‐specific PCR amplification and Sanger sequencing [19].

Our strategy enabled detection of a novel 1.5 kb deletion in Xq28, a representative CNV/SV, which turned out to be highly polymorphic, even prevalent in our general population (> 80%).

An additional potential application of the approach would be the structural characterisation of highly homologous duplicated loci (e.g., *SMN1*/*SMN2*, *CYP21A2*/*CYP21A1P* or *GBA*/*GBAP1*), where conventional short‐read sequencing frequently encounters mapping ambiguities. Although this possibility remains to be experimentally evaluated, the successful identification of an inversion mediated by 99% identical 8.4‐kb IRs suggests that the LPER strategy may provide additional discriminatory information in these complex genomic regions.

To demonstrate its ability to detect SNV/indels with potential to impact gene function (e.g., deep‐intronic variants), the application of small‐variant calling algorithms on our mapped read dataset indicated a nonpathogenic nucleotide transition (C > T) on the polypyrimidine tract of the splicing acceptor site of *F8*‐intron 3, which was confirmed.

It is clear that our system represents an advance over Illumina′s short‐read WGS platform, extending haplotyping from conventional PERs of 350 bp to *Bcl*I LPERs averaging 4 kb, ranging from < 0.5 to > 25 kb (Figure 3B). The comparison of our system with long‐read WGS platforms (e.g., PacBio and Oxford Nanopore Technology ONT) is likely to be disadvantageous for detection and genotyping of large SVs, although the scarce literature comparing short‐ versus long‐reads platforms based on specific experimental data (e.g., Inv22 genotyping in Haemophilia A). The comparison between the different approaches for massive parallel sequencing (MPS) (i.e., short‐read Illumina platform, long‐read PacBio and ONT and the so‐called pseudo long‐read) indicates differences in DNA input requirements, costs, capacity to detect and characterise small variants and SVs, current and prospective clinical applicability and reliability of the results.

As indicated previously, short‐read WGS exceeds all long‐read platforms in terms of accuracy for SNV/indels genotyping, constituting the gold standard, even superior to the PacBio HiFi platform (99.9%) and even more so to the ONT (92%–99%). Depending on the quality of the DNA input, both PacBio and ONT can extend sequence tracts in the range of 4– > 50 kb, exceeding the maximum lengths of full‐length LINE elements. It is worth noting that the ligation round associated with the formation of restriction‐circles in our approach functions as a nick‐sealing round, improving the integrity/quality of the DNA input.

Conventional short‐read WGS constitutes the most economical approach and is widely used in clinical genetics around the world, although in terms of the capacity to genotype SVs as well as SNV/indels with high precision, and despite being the most expensive technology, PacBio HiFi is a good future alternative for gene testing laboratories, worldwide. Regarding pseudo long‐read platforms (e.g., Bionano or 10x Genomics), which use optical cytomolecular maps (not DNA sequencing), or short‐read sequencing of physically separated large DNA molecules, they require high‐quality or ultra long DNA such as long‐read platforms, involved intermediate costs, and their specific capabilities do not fulfil the requirement for their direct application in clinical genetics.

The genotyping of large perfect inversions merits a focused discussion. The introduction of long‐read NGS technologies (ONT, PacBio platforms) generated an advance in the characterisation of SVs, especially large CNVs, and theoretically, also genomic inversions [36–38]. However, large perfect inversions still remain extremely problematic to resolve even for these technologies. A paradigmatic example of these limitations is the need to input inversion‐specific long‐range PCR amplicons as substrates for long‐read NGS, for example, for detection of Inv22 causing half of patients with severe‐HA [39–41]. To the best of our knowledge, there have been no reports of massive detection of the Inv22 by second‐ or even third‐generation NGS [42].

To illustrate this challenge, coagulation NGS‐panels available [43, 44] need to be complemented with an out‐board investigation of *F8*‐Inv22 by specific methods, such as long‐range [45] or IS‐PCR [18]. Likewise, the NGS‐based haemophilia genotyping initiative in the US (My Life, Our Future) applies a panel of molecular inversion probes covering the *F8* and *F9* most relevant regions but also targeting the relevant Inv22 and Inv1 restriction circles [46].

Significantly, our findings sugested that our approach can detect Inv22 and Inv1 by second‐generation NGS in an untargeted manner through the recognition of Normal/reference LPERs associated with the *int22h* and *int1h* duplicons.

Undoubtedly, the most significant prediction of our practical approach was the finding of a 1.2 kb perfect inversion, mediated by non‐allelic recombination between highly homologous (99.6% identical) 8.4 kb‐long sequences, which remained undetectable by conventional SV‐calling algorithms. By considering the mechanism of NAHR characterised by reciprocal‐crossovers equally located within the IR, this inversion is defined as g.(153066014_153074418)_(153075613_153084041)inv. Xq28/1.2 kb‐inv resulted polymorphic in the general population with an AF of one‐third. The combination of a precise molecular mechanism related to most genomic recombination events and the experimental complications of detecting them in practice raises the hypothesis that already identified perfect inversions represents the visible tip of the iceberg of a much prevalent undetected population in the HG.

In conclusion, the practical utility of our approach relies on the comprehensive typeset of variant calling, including deep‐intronic variants, SNV/indels and SVs (such as perfect inversions), and it will be improved by using a robust capture system for specific restriction‐circles on selected genes.

A limitation of our approach relates to the specificity of the restriction map: rearrangements confined into a fragment will not generate informative LPERs. A change in the RE may generate another pattern to enhance informativity. The bioinformatics associated with SV‐calling using LPER operator was simplified by use of complete digestion of the RE. All massive genomic analyses including NGS/WGS are used as a variant screening tool in medical genetics and it should always be accompanied by an independent gene‐testing experiment.

Grounded on the findings presented in this work, we believe that our integrated strategy, based on short‐read WGS of restriction‐circles and a pipeline combining novel and conventional variant‐calling algorithms, has potential to be successfully applied in genomic research and clinical setting worldwide.

**Nomenclature**
AF: allelic frequency
AWK: Aho–Weinberger–Kernighan programming language
BAM: binary alignment map
BED: browser extensible data
BND: break end
BWA: Burrows–Wheeler Aligner
CIGAR: concise idiosyncratic gapped alignment report
CNV: copy number variant
CTAB: cetyl‐trimethyl‐ammonium bromide
DEL: deletion
DUP: duplication
GATK: Genome Analysis Toolkit
gDNA: genomic DNA
HG: human genome
HGVS: Human Genome Variation Society
INDELs: insertions–deletions
INS: nontandem duplications
INV(INS): inverted insertions
INV: inversion
IR: inverted repeat
IS‐PCR: inverse shifting‐PCR
LCR: low copy number repeats
LINE: long interspersed nuclear elements
LPER: linking‐paired‐end read
MAPQ: map quality
MLPA: multiplex ligation‐dependent probe amplification
MPS: massive parallel sequencing
N/A: not assigned
NAHR: nonallelic homologous recombination
NGS: next‐generation sequencing
NORM: REF: normal, reference genomic structure
PBL: peripheral blood leukocytes
PER: paired‐end read
RE: restriction enzymes
RFLP: restriction fragment length polymorphism
RS: restriction recognition site
SAM: sequence alignment map
SINE: short interspersed nuclear element
SNP: single nucleotide polymorphism
SNP‐RFLP: single nucleotide polymorphism‐restriction fragment length polymorphism
SNV: single nucleotide variant
SVs: structural variants
TSV: tab separated value
VCF: variant call format
VEP: Variant Effect Predictor
WGS: whole‐genome sequencing

## Author Contributions

F.G. and C.D.D.B. have equally contributed to this paper and should be considered as last authors.

## Funding

This study was supported by grants from the National Research Council (CONICET) and the National Agency for Scientific and Technological Promotion (ANPCyT).

## Conflicts of Interest

The authors declare no conflicts of interest.

## Supporting Information

Additional supporting information can be found online in the Supporting Information section.

## Supporting information


**Supporting Information 1** Figure S1: Characteristics of reads forming linking‐PERs (LPERs).

**Supporting Information 2** Figure S2: Rationale of structural variants′ (SV) molecular diagnosis.

**Supporting Information 3** Figure S3: DEL polymorphism analysis.

**Supporting Information 4** Figure S4: Analysis of the inverted repeat (IR) involved in Xq28 inversion.

**Supporting Information 5** Figure S5: INV polymorphism analysis.

**Supporting Information 6** Figure S6: *F8* intron 22 inversion analysis.

**Supporting Information 7** Figure S7: Experimental confirmation of the single nucleotide variant (SNV) rs35621875.

**Supporting Information 8** Table S1: Filtered LPERs for X‐linked DELs, DUPs and INVs.

**Supporting Information 9** Table S2: Lumpy, Delly, SvABA SV detection.

**Supporting Information 10** Table S3: BclI LPERs, Dx no‐INV22.

**Supporting Information 11** Table S4: *F8* small variant call.

**Supporting Information 12** Text S1: Additional materials and methods (bioinformatic‐pipeline details).

**Supporting Information 13** Text S2: Linux bash script‐SV detection.

**Supporting Information 14** Text S3: DMD SVs coordinates calculation.

## Data Availability

The data that support the findings of this study are available on request from the corresponding author. The data are not publicly available due to privacy or ethical restrictions.
