# Supplementary material for: Novel Strategy for Structural Variant Genotyping by Short‐Read Genomic Sequencing From Restriction‐Circles: Experimental and Bioinformatics Proof‐of‐Concept
Source: Hum Mutat. 2026 Oct 1;2026:2169350. doi: 10.1155/humu/2169350 (PMC13628565; doi:10.1155/humu/2169350)
Supplement: Supplementary file 1 [file HUMU-2026-2169350-s002.docx]

**A.**

**153065931**

**Xq28**

**153075234**

**153084298**

**8404 bp**

**8428 bp**

***PNMA6A***

**Xq-tel**

**X-cen**

**153066014**

**153074418**

**153075613**

**153084041**

**153077014**

***PNMA6B Pseudogene***

**IR**

**Telomeric Arm**

**IR**

**Centromeric Arm**

**B**

**B**

**B**

**B.**

**BLAST alignment. Inverted Repeat (IR) Characteristics.**

**NC_000023.11 Homo sapiens chromosome X, GRCh38.p14**

```
Score	             Expect	  Identities	       Gaps	       Strand
15367 bits (8321)   0.0	  8397/8429(99%)	24/8429(0%)	Plus/Minus


153066014  CTCCTCCAGAGGGACTTGACTTCAATGAGTTGAAGACTGTGCCACAGGCAACCAGCTGTG
           ||||||||||||||||||||||||||||||||||||||||||||||||||||||||||||
153084041  CTCCTCCAGAGGGACTTGACTTCAATGAGTTGAAGACTGTGCCACAGGCAACCAGCTGTG

153066074  TCAAGGACCAACGCAGAACAAGCTCACACTTATTCAGCCCAGCAGGACCCTGAGTCTGGG
           ||||||||||||||||||||||||||||||||||||||||||||||||||||||||||||
153083981  TCAAGGACCAACGCAGAACAAGCTCACACTTATTCAGCCCAGCAGGACCCTGAGTCTGGG

153066134  AAGCATGGCGTGCCACATCGGGTTGTCCCTCAGAAAAACCCTGCTAGGTGGGAAGGAGCC
           ||||||||||||||||||||||||||||||||||||||||||||||||||||||||||||
153083921  AAGCATGGCGTGCCACATCGGGTTGTCCCTCAGAAAAACCCTGCTAGGTGGGAAGGAGCC

153066194  ACCTGCACCTGTGAGGCTGCGGAGGACAGGCTAGGGGCAGGGCAGGTTGTACGTGCATGT
           ||||||||||||||||||||||||||||||||||||||||||||||||||||||||||||
153083861  ACCTGCACCTGTGAGGCTGCGGAGGACAGGCTAGGGGCAGGGCAGGTTGTACGTGCATGT

153066254  GCATGCGTGCGTGTGCGTGCGTGCGTGCGTGTGTGTGTGTGTGT--------TTCCGTGT
           ||||||||||||||||||||||||||||||||||||||||||||        ||| ||||
153083801  GCATGCGTGCGTGTGCGTGCGTGCGTGCGTGTGTGTGTGTGTGTGTGTGTGTTTCAGTGT

153066306  GTGTGTGGGTGGGGCACAGCCAGACAGAAGCTGAGGAAGTACTGCTCCCCCCACCCCAGG
           ||||||||||||||||||||||||||||||||||||||||||||||||||||||||||||
153083741  GTGTGTGGGTGGGGCACAGCCAGACAGAAGCTGAGGAAGTACTGCTCCCCCCACCCCAGG

153066366  TAGGGGCAGCCAGCCAGGGAATGGCAGGGTCATATGGCAGGAGGCAGGTGCTAGCCTGGC
           ||||||||||||||||||||||||||||||||||||||||||||||||||||||||||||
153083681  TAGGGGCAGCCAGCCAGGGAATGGCAGGGTCATATGGCAGGAGGCAGGTGCTAGCCTGGC

153066426  AGCCCAAGGACCTGTCGTGGGCTCTGGGCAGGGCAGAGAGCCGTCGGGAGCAGAATCAAA
           ||||||||| ||||||||||||||||||||||||||||||||||||||||||||||||||
153083621  AGCCCAAGGGCCTGTCGTGGGCTCTGGGCAGGGCAGAGAGCCGTCGGGAGCAGAATCAAA

153066486  GAGGGCATTGGAGGAAGAAGCTTCTGAGCAGTCAGAGCAGGCCGCGGCCGGTGGGGAGCT
           ||||||||||||||||||||||||||||||||||||||||||||||||||||||||||||
153083561  GAGGGCATTGGAGGAAGAAGCTTCTGAGCAGTCAGAGCAGGCCGCGGCCGGTGGGGAGCT

153066546  GGCCCGAAGCCACAGACTGGGGAGCTTTGGCCCTGCCTGAGGTAGGACGGTAGGGAGGAG
           ||||||||||||||||||||||||||||||||||||||||||||||||||||||||||||
153083501  GGCCCGAAGCCACAGACTGGGGAGCTTTGGCCCTGCCTGAGGTAGGACGGTAGGGAGGAG
153066606  CCCCAGGCTCTCCCTGGAGGACACAAGGGGGCCCCCGTTGGCTGTGCAGAGCCTGGGCCC
           ||||||||||||||||||||||||||||||||||||||||||||||||||||||||||||
153083441  CCCCAGGCTCTCCCTGGAGGACACAAGGGGGCCCCCGTTGGCTGTGCAGAGCCTGGGCCC

153066666  TCGGACATTGTGGTGCACAGCAAAGAAGCCCTTCTTGGTTCAAGAGCAGACAATGCTCCC
           ||||||||||||||||||||||||||||||||||||||||||||||||||||||||||||
153083381  TCGGACATTGTGGTGCACAGCAAAGAAGCCCTTCTTGGTTCAAGAGCAGACAATGCTCCC

153066726  ACTCAACCTGTGGAGGCTGTAAGGGACACGTCCGAGGTCAGCCAGCTGGGAGGCAACCAG
           ||||||||||||||||||||||||||||||||||||||||||||||||||||||||||||
153083321  ACTCAACCTGTGGAGGCTGTAAGGGACACGTCCGAGGTCAGCCAGCTGGGAGGCAACCAG

153066786  GTGGGGAGAAACACACACGGAGACCTCCTCTGCGTCCCGGCCTGCGTTGGAGGCACTCAG
           ||||||||||||||||||||||||||||||||||||||||||||||||||||||||||||
153083261  GTGGGGAGAAACACACACGGAGACCTCCTCTGCGTCCCGGCCTGCGTTGGAGGCACTCAG

153066846  CAAGGCTGGCTGTCCTTGGGACTTGTTTCTCAAGAGCCAGTACTACAGCAGGTGCTCTAC
           ||||||||||||||||||||||||||||||||||||||||||||||||||||||||||||
153083201  CAAGGCTGGCTGTCCTTGGGACTTGTTTCTCAAGAGCCAGTACTACAGCAGGTGCTCTAC

153066906  GGAGGCGTCTGGTCACCAAGCAACCCAGAGCCTTCTGTGAATCTTGATTGCAGCATCTCC
           ||||||||||||||||||||||||||||||||||||||||||||||||||||||||||||
153083141  GGAGGCGTCTGGTCACCAAGCAACCCAGAGCCTTCTGTGAATCTTGATTGCAGCATCTCC

153066966  AATCAAGTGCCACCTCACAGTACAGTGCTTCCGTGTTTATCCCTTCAGGTGTCCAACATC
           ||||||||||||||||||||||||||||||||||||||||||||||||||||||||||||
153083081  AATCAAGTGCCACCTCACAGTACAGTGCTTCCGTGTTTATCCCTTCAGGTGTCCAACATC

153067026  TGCTGGGCACGCTGGCTCTGTGCAGTGCCTTCTGCTGGCAATCAGGGGGACCAAGCCAAA
           ||||||||||||||||||||||||||||||||||||||||||||||||||||||||||||
153083021  TGCTGGGCACGCTGGCTCTGTGCAGTGCCTTCTGCTGGCAATCAGGGGGACCAAGCCAAA

153067086  AAGATGGAAGCCTCTGGGAGCCAAACCAAGCAGGGGCCACCTATACAATTCCTCTACCTT
           ||||||||||||||||||||||||||||||||||||||||||||||||||||||||||||
153082961  AAGATGGAAGCCTCTGGGAGCCAAACCAAGCAGGGGCCACCTATACAATTCCTCTACCTT

153067146  TACCAGGGAGTCTGCCTGCCCACAGGGCAGGGCTGGGTGCTGCGGGGCAGGGTGGGGAGG
           ||||||||||||||||||||||||||||||||||||||||||| ||||||||||||||||
153082901  TACCAGGGAGTCTGCCTGCCCACAGGGCAGGGCTGGGTGCTGCAGGGCAGGGTGGGGAGG

153067206  GACGGGAAGATGGAGAGTGGCAGGCTGGCGGGAGGGAGGAAGAGGGCAGAGCGAATGTCT
           ||||||||||||||||||||||||||||||||||||||||||||||||||||||||||||
153082841  GACGGGAAGATGGAGAGTGGCAGGCTGGCGGGAGGGAGGAAGAGGGCAGAGCGAATGTCT

153067266  ACCTGGGTGTCACACGAGCCACTTGAATGTGACCACGGCCACAGGAACTCATCCCCATGC
           ||||||||||||||||||||||||||||||||||||||||||||||||||||||||||||
153082781  ACCTGGGTGTCACACGAGCCACTTGAATGTGACCACGGCCACAGGAACTCATCCCCATGC

153067326  CCGTCACCCCTCTTTCCACAAAAGGCAAGAGGAAAGACAACTGCCAGCATGATCCCCATG
           ||||||||||||||||||||||||||||||||||||||||||||||||||||||||||||
153082721  CCGTCACCCCTCTTTCCACAAAAGGCAAGAGGAAAGACAACTGCCAGCATGATCCCCATG

153067386  TCAGACAAGGGTCCTGCCTTCCACCCGGTCCACACACAACAAGGAACCCGGGACCCTGCC
           ||||||||||||||||||||||||||||||||||||||||||||||||||||||||||||
153082661  TCAGACAAGGGTCCTGCCTTCCACCCGGTCCACACACAACAAGGAACCCGGGACCCTGCC

153067446  TGAGCCCTCCCCGGGCCTTCTCCCTCTCTAGAGCCTGTATCTTCTGCTTTCCCTTAGACC
           ||||||||||||||||||||||||||||||||||||||||||||||||||||||||||||
153082601  TGAGCCCTCCCCGGGCCTTCTCCCTCTCTAGAGCCTGTATCTTCTGCTTTCCCTTAGACC

153067506  CTCCTTGCCCAGCCCCGGGGTCGGGGGGGCCTGGACTTGGGCTGCAGCCCCCTCGCTGCT
           ||||||||||||||||||||||||||||||||||||||||||||||||||||||||||||
153082541  CTCCTTGCCCAGCCCCGGGGTCGGGGGGGCCTGGACTTGGGCTGCAGCCCCCTCGCTGCT
153067566  CCAGGCTTCCCATCCTGCTGATTAGTTCCCCCAGACGTCTGGCCGGCCGGTCTCAGCATC
           ||||||||||||||||||||||||||||||||||||||||||||||||||||||||||||
153082481  CCAGGCTTCCCATCCTGCTGATTAGTTCCCCCAGACGTCTGGCCGGCCGGTCTCAGCATC

153067626  ACCACCTCCTGGCTTCAAACCCATCGGGGCTCCCCACAGCCCTCAGGATAAGGTCCCGGT
           ||||||||||||||||||||||||||||||||||||||||||||||||||||||||||||
153082421  ACCACCTCCTGGCTTCAAACCCATCGGGGCTCCCCACAGCCCTCAGGATAAGGTCCCGGT

153067686  ACCCTGGCAGGCTGGAGCAGGCTGACAGATTCAGGCACTTGCTGGAAAGAAGGCACCGAG
           ||||||||||||||||||||||||||||||||||||||||||||||||||||||||||||
153082361  ACCCTGGCAGGCTGGAGCAGGCTGACAGATTCAGGCACTTGCTGGAAAGAAGGCACCGAG

153067746  AGCCTCCTCTGGGCCACACTCTGTTCTAGGTGCTGGGGATCTCAGGGTAGGAAACGCTGT
           ||||||||||||||||||||||||||||||||||||||||||||||||||||||||||||
153082301  AGCCTCCTCTGGGCCACACTCTGTTCTAGGTGCTGGGGATCTCAGGGTAGGAAACGCTGT

153067806  ACTACGGTTTGAACGTTGTGTCCCTGCCAAAATTCATGTTGAAAGTTAGTCCCTAACGCA
           ||||||||||||||||||||||||||||||||||||||||||||||||||||||||||||
153082241  ACTACGGTTTGAACGTTGTGTCCCTGCCAAAATTCATGTTGAAAGTTAGTCCCTAACGCA

153067866  GCAGTGTTAAGGGGTGAGACCTTTAGGAGGCAATGAGACCATGGGGGCAGAGCCCTCGGG
           ||||||||||||||||||||||||||||||||||||||||||||||||||||||||||||
153082181  GCAGTGTTAAGGGGTGAGACCTTTAGGAGGCAATGAGACCATGGGGGCAGAGCCCTCGGG

153067926  GATGAGAGGAGTCCCTTGTAAAAGGCTGGAAAGCACTAGGTAAGCCCCCTTTTTGCTGTT
           ||||||||||||||||||||||||||||||||||||||||||||||||||||||||||||
153082121  GATGAGAGGAGTCCCTTGTAAAAGGCTGGAAAGCACTAGGTAAGCCCCCTTTTTGCTGTT

153067986  CCCTCCCCACGACCATGTGAGGACGCAGGCACAAGACACCATCTTGGAAGGAGAGACTGA
           |||||||||||||||||||||||| |||||||||||||||||||||||||||||||||||
153082061  CCCTCCCCACGACCATGTGAGGACACAGGCACAAGACACCATCTTGGAAGGAGAGACTGA

153068046  GCCCCCACCAGATGCTGAACCTACTGGTGCCTTGATCTTGGGCTTCCCAGCCTCCGGAAT
           ||||||||||||||||||||||||||||||||||||||||||||||||||||||||||||
153082001  GCCCCCACCAGATGCTGAACCTACTGGTGCCTTGATCTTGGGCTTCCCAGCCTCCGGAAT

153068106  TGGGAACAAGAAATTTCTGTTCTTTTGTAAGTTTCCCAGTCTCCAGTGCCTTGACTTTGA
           ||||||||||||||||||||||||||||||||||||||||||||||||||||||||||||
153081941  TGGGAACAAGAAATTTCTGTTCTTTTGTAAGTTTCCCAGTCTCCAGTGCCTTGACTTTGA

153068166  GCTTCCCAGCCCCCAGAATTGGGAGCAATACATTTCTGTTCTTTGCAAGTTACCCAGTCT
           ||||||||||||||||||||||||||||||||||||||||||||||||||||||||||||
153081881  GCTTCCCAGCCCCCAGAATTGGGAGCAATACATTTCTGTTCTTTGCAAGTTACCCAGTCT

153068226  CAGGTATTTTGCTATAGCAGTGCAAATGGGCTAGACACACAGGCAAGTCCACTCCACCTT
           |||||||||||||||||||||||||||||||||||||||||||||||||||| |||||||
153081821  CAGGTATTTTGCTATAGCAGTGCAAATGGGCTAGACACACAGGCAAGTCCACCCCACCTT

153068286  CTGGGGCTCTAGCTTGCCACCTGGGGAGGGAGCCCCTGATACCCAAAAGCAAAGACTCAG
           ||||||||||||||||||||||||||||||||||||||||||||||||||||||||||||
153081761  CTGGGGCTCTAGCTTGCCACCTGGGGAGGGAGCCCCTGATACCCAAAAGCAAAGACTCAG

153068346  ACCAGCATTTCTCACGTGCCGACACATGTACTGATGCCGCCAATAAGAATATAAACCGAG
           ||||||||||||||||||||||||||||||||||||||||||||||||||||||||||||
153081701  ACCAGCATTTCTCACGTGCCGACACATGTACTGATGCCGCCAATAAGAATATAAACCGAG

153068406  GGCACGTGATTTGTGTGTCCCAGCAGAGGGGGCCTATGGGAGGAGGGGACAGTTCAGCTG
           ||||||||||||||||||||||||||||||||||||||||||||||||||||||||||||
153081641  GGCACGTGATTTGTGTGTCCCAGCAGAGGGGGCCTATGGGAGGAGGGGACAGTTCAGCTG

153068466  AGACATGAAGGAGGAGGAGGTGCAGGAGGAGGGGGAGGAGGAGGA---------------
           |||||||||||||||||||||||||||||||||||||||||||||
153081581  AGACATGAAGGAGGAGGAGGTGCAGGAGGAGGGGGAGGAGGAGGAGGGGGAGGAGGAGGA
153068511  ATAGGAGGAGGAGGAGGAGGAGGGAGAGGAAGAGCAGGAGGAAGCTGCTGCCACAGAAGG
           ||||||||||||||||||||||||||||||||||||||||||||||||||||||||||||
153081521  ATAGGAGGAGGAGGAGGAGGAGGGAGAGGAAGAGCAGGAGGAAGCTGCTGCCACAGAAGG

153068571  TTCTTGGGCCTGGATGGCTCACACAGAGGAAGGAGGGATGGCAAAGGCCGAAGGTGGGAA
           ||||||||||||||||||||||||||||||||||||||||||||||||||||||||||||
153081461  TTCTTGGGCCTGGATGGCTCACACAGAGGAAGGAGGGATGGCAAAGGCCGAAGGTGGGAA

153068631  CAAGCCCTGTGCTTTGGGGCCAGCTGAACAGGCCAGATCGGCAGGAATGTGGTGAGAGGA
           ||||||||||||||||||||||||||||||||||||||||||||||||||||||||||||
153081401  CAAGCCCTGTGCTTTGGGGCCAGCTGAACAGGCCAGATCGGCAGGAATGTGGTGAGAGGA

153068691  GCTGGGGGCATGGCAACATGGGGTGGATTTCGGGGAAGGAAGGAGAGCATCAGAGGAAGC
           ||||||||||||||||||||||||||||||||||||||||||||||||||||||||||||
153081341  GCTGGGGGCATGGCAACATGGGGTGGATTTCGGGGAAGGAAGGAGAGCATCAGAGGAAGC

153068751  CAGATCCCCAGGAGCAGCGTGGGGGGTGCTCTTGACCAGGATGGGGACTTTGAGATTACT
           ||||||||||||||||||||||||||||||||||||||||||||||||||||||||||||
153081281  CAGATCCCCAGGAGCAGCGTGGGGGGTGCTCTTGACCAGGATGGGGACTTTGAGATTACT

153068811  CTAAGGAGATGGGGTGCCACAGGGAGGAGCAGGGTCTGGTGGTGTTGCAAAAAGATCACC
           ||||||||||||||||||||||||||||||||||||||||||||||||||||||||||||
153081221  CTAAGGAGATGGGGTGCCACAGGGAGGAGCAGGGTCTGGTGGTGTTGCAAAAAGATCACC

153068871  CTGGCTGCTGAGGGGGTGTTGGTGGAGCAGGGAGGAAGCGGAGTGGAGGCAGGGAGAACA
           ||||||||||||||||||||||||||||||||||||||||||||||||||||||||||||
153081161  CTGGCTGCTGAGGGGGTGTTGGTGGAGCAGGGAGGAAGCGGAGTGGAGGCAGGGAGAACA

153068931  GGAGGCAGGGCGGGCCAAGGACCACGGAGATGAGAAGGGTAGCAGTCTTGGAGCAGAGTC
           ||||||||||||||||||||||||||||||||||||||||||||||||||||||||||||
153081101  GGAGGCAGGGCGGGCCAAGGACCACGGAGATGAGAAGGGTAGCAGTCTTGGAGCAGAGTC

153068991  AACAAAATCCAATGATGGGTTAGATTTGGGAGTGAGGAACAAAGGAGAGGGGAGAGAATC
           ||||||||||||||||||||||||||||||||||||||||||||||||||||||||||||
153081041  AACAAAATCCAATGATGGGTTAGATTTGGGAGTGAGGAACAAAGGAGAGGGGAGAGAATC

153069051  TTGGCTGCTTTGTTTGAGTCACCAGGTAGGAGGCAGAGCCCTTTGCACAGAGGGGGAAGC
           ||||||||||||||||||||||||||||||||||||||||||||||||||||||||||||
153080981  TTGGCTGCTTTGTTTGAGTCACCAGGTAGGAGGCAGAGCCCTTTGCACAGAGGGGGAAGC

153069111  CCGGGGACAGTGTAGGCATGGTGAAGGGGACGAAGCCCTCCGGTTTGAACATCTTAGCTC
           ||||||||||||||||||||||||||||||||||||||||||||||||||||||||||||
153080921  CCGGGGACAGTGTAGGCATGGTGAAGGGGACGAAGCCCTCCGGTTTGAACATCTTAGCTC

153069171  TGAAATGTCTGCGGGGCATCCACGTGGGCAGATGGATGGGTACGTGCGGAGCTTGGAAGA
           ||||||||||||||||||||||||||||||||||||||||||||||||||||||||||||
153080861  TGAAATGTCTGCGGGGCATCCACGTGGGCAGATGGATGGGTACGTGCGGAGCTTGGAAGA

153069231  GAGGTCACGTCCAGAGACAGAGCATTCATCGGAGGTGCCGTATGTCCCAGGGTTGAGGCT
           ||||||||||||||||||||||||||||||||||||||||||||||||||||||||||||
153080801  GAGGTCACGTCCAGAGACAGAGCATTCATCGGAGGTGCCGTATGTCCCAGGGTTGAGGCT

153069291  GGGAGCCCGCCCCCACCACGCTTAGATCCAGCAAAGTAGGAGGAGGAGGAGGAAGGTCCA
           ||||||||||||||||||||||||||||||||||||||||||||||||||||||||||||
153080741  GGGAGCCCGCCCCCACCACGCTTAGATCCAGCAAAGTAGGAGGAGGAGGAGGAAGGTCCA

153069351  AGGGCAGAGAGGCAGGTAGAGAGGGAGGAGGTCAGCCCAAAAGTGGGGTGGGGGAGAGGA
           ||||||||||||||||||||||||||||||||||||||||||||||||||||||||||||
153080681  AGGGCAGAGAGGCAGGTAGAGAGGGAGGAGGTCAGCCCAAAAGTGGGGTGGGGGAGAGGA

153069411  AGAAGCTGAGAAAGGAAATGGTTTTGAGAAGGAAGGAGAGTCAACTGTGTTGAGAGGTGG
           ||||||||||||||||||||||||||||||||||||||||||||||||||||||||||||
153080621  AGAAGCTGAGAAAGGAAATGGTTTTGAGAAGGAAGGAGAGTCAACTGTGTTGAGAGGTGG
153069471  AGACAATGGGGACAGAGGACTGACAAAAGAAAGACAGTTGACGTGTCACAGCCCAGCCTT
           ||||||||||||||||||||||||||||||||||||||||||||||||||||||||||||
153080561  AGACAATGGGGACAGAGGACTGACAAAAGAAAGACAGTTGACGTGTCACAGCCCAGCCTT

153069531  GCCCGGGGTTGGCGGGGGGCAATGGGTGTCCTGCAGCGTCCTGGACTTGGAGTCAGCAGG
           ||||||||||||||||||||||||||||||||||||||||||||||||||||||||||||
153080501  GCCCGGGGTTGGCGGGGGGCAATGGGTGTCCTGCAGCGTCCTGGACTTGGAGTCAGCAGG

153069591  TGCAGAAGGAAGGAGAGTGGGAAGAGACAAGGCCGAAAACAGGAAATTAGTTGCGAGCCG
           ||||||||||||||||||||||||||||||||||||||||||||||||||||||||||||
153080441  TGCAGAAGGAAGGAGAGTGGGAAGAGACAAGGCCGAAAACAGGAAATTAGTTGCGAGCCG

153069651  GCCGGTGGGCTGGCTGGGCCAGTTGGAGACACCAGACCCACAGATGTTCCCAGGGCGGGG
           ||||||||||||||||||||||||||||||||||||||||||||||||||||||||||||
153080381  GCCGGTGGGCTGGCTGGGCCAGTTGGAGACACCAGACCCACAGATGTTCCCAGGGCGGGG

153069711  GCGGCCGAGGCGGGGGGGGGG-AGGGGGGGGGGGTCGGTGCCCTTTCTGAAGAAGGACTC
           ||||||||||||||||||||| ||||||||||||||||||||||||||||||||||||||
153080321  GCGGCCGAGGCGGGGGGGGGGGAGGGGGGGGGGGTCGGTGCCCTTTCTGAAGAAGGACTC

153069770  CAGAAAAATCACGGCTGAAAACAAGAGAGAGGACAATATGTGGTATAAGCCGTGACAAGT
           ||||||||||||||||||||||||||||||||||||||||||||||||||||||||||||
153080261  CAGAAAAATCACGGCTGAAAACAAGAGAGAGGACAATATGTGGTATAAGCCGTGACAAGT

153069830  CTAACACTAAAGGTTCGATAGACAGACACTCTCACAGCAGAGGTAAAGAGCAGCAGTTTG
           ||||||||||||||||||||||||||||||||||||||||||||||||||||||||||||
153080201  CTAACACTAAAGGTTCGATAGACAGACACTCTCACAGCAGAGGTAAAGAGCAGCAGTTTG

153069890  CCTGAATGAGAACAAGCAGTCTTGGCCCTCAAGGGGGAGAAGCGGTGGGGGGCAGGGGGG
           ||||||||||||||||||||||||||||||||||||||||||||||||||||||||||||
153080141  CCTGAATGAGAACAAGCAGTCTTGGCCCTCAAGGGGGAGAAGCGGTGGGGGGCAGGGGGG

153069950  CGGGGAGGACTGGACACACGAGCCCAACCGGGGAGGCTGCTTTGTGACAGAAGACATGCG
           ||||||||||||||||||||||||||||||||||||||||||||||||||||||||||||
153080081  CGGGGAGGACTGGACACACGAGCCCAACCGGGGAGGCTGCTTTGTGACAGAAGACATGCG

153070010  TTTCTTCGCTGACCCCACGTTTACTACCTGGGAGGTAGAAAAGCCTGGGGAGCAAGACCA
           ||||||||||||||||||||||||||||||||||||||||||||||||||||||||||||
153080021  TTTCTTCGCTGACCCCACGTTTACTACCTGGGAGGTAGAAAAGCCTGGGGAGCAAGACCA

153070070  TGCCAACCTAAGTACCACACAGAGAGGGAGCCTCCCTATAAGAATGGTATTCAGAAATAG
           ||||||||||||||||||||||||||||||||||||||||||||||||||||||||||||
153079961  TGCCAACCTAAGTACCACACAGAGAGGGAGCCTCCCTATAAGAATGGTATTCAGAAATAG

153070130  GCACTGCAGTGGGGACGCGTGCGCCCTAGTGAACTATGTGCATATTCAGGCAGGGGAAGG
           ||||||||||||||||||||||||||||||||||||||||||||||||||||||||||||
153079901  GCACTGCAGTGGGGACGCGTGCGCCCTAGTGAACTATGTGCATATTCAGGCAGGGGAAGG

153070190  CAGACAAAGGTTTTTCAGGACAAGTGAAGAGGATTACATAATTGTTTTGACATAATGATC
           ||||||||||||||||||||||||||||||||||||||||||||||||||||||||||||
153079841  CAGACAAAGGTTTTTCAGGACAAGTGAAGAGGATTACATAATTGTTTTGACATAATGATC

153070250  TTTGGCTACAAGGATGAATAACAAGGGTAGCATTGGTCAGGGGCTTGGACAGGAAGTTGC
           ||||||||||||||||||||||||||||||||||||||||||||||||||||||||||||
153079781  TTTGGCTACAAGGATGAATAACAAGGGTAGCATTGGTCAGGGGCTTGGACAGGAAGTTGC

153070310  TGGGCAGGTGTCCTCACAGAAGTATTATTTGTGTCAGGTTGCAACAGCCTTTGTGCAAAT
           ||||||||||||||||||||||||||||||||||||||||||||||||||||||||||||
153079721  TGGGCAGGTGTCCTCACAGAAGTATTATTTGTGTCAGGTTGCAACAGCCTTTGTGCAAAT

153070370  TTGTGGGTTTTGCAGTCTTCCGTGATAGCTTTTGTTATCAGGCATTCGTGCATGGGAACC
           ||||||||||||||||||||||||||||||||||||||||||||||||||||||||||||
153079661  TTGTGGGTTTTGCAGTCTTCCGTGATAGCTTTTGTTATCAGGCATTCGTGCATGGGAACC
153070430  CTGCCTAGGTGGCCCTCCACAGCTCTATTTGTCAGGTTTTTGGATTTTTGTTTGCTTGAA
           ||||||||||||||||||||||||||||||||||||||||||||||||||||||||||||
153079601  CTGCCTAGGTGGCCCTCCACAGCTCTATTTGTCAGGTTTTTGGATTTTTGTTTGCTTGAA

153070490  CAACAAGTGACTCCATCTTGATTCTGAAACCTTTCACAACCATACGGGCAAGCCAAAGTC
           ||||||||||||||||||||||||||||||||||||||||||||||||||||||||||||
153079541  CAACAAGTGACTCCATCTTGATTCTGAAACCTTTCACAACCATACGGGCAAGCCAAAGTC

153070550  TAATTGTGAGGCCCACTCCAATGCGACCTGCTTCTGGGCAACGTGATAGACTGGCCATGG
           ||||||||||||||||||||||||||||||||||||||||||||||||||||||||||||
153079481  TAATTGTGAGGCCCACTCCAATGCGACCTGCTTCTGGGCAACGTGATAGACTGGCCATGG

153070610  CTTATATCACAAAAGGAGAAAGGCTTCGATCTTGACGCTAGGAACTTTCTCCTGGAAACG
           ||||||||||||||||||||||||||||||||||||||||||||||||||||||||||||
153079421  CTTATATCACAAAAGGAGAAAGGCTTCGATCTTGACGCTAGGAACTTTCTCCTGGAAACG

153070670  GGCCGATGACGCCTGTCATCTGCGAGATGAGGAGGAAAGGAGCAAAGGAGATTCACAGAA
           |||| |||||||||||||||||||||||||||||||||||||||||||||||||||||||
153079361  GGCCAATGACGCCTGTCATCTGCGAGATGAGGAGGAAAGGAGCAAAGGAGATTCACAGAA

153070730  GAGGAACCAGGAACGGCCAGTGAGAGGATGCACGACCTCGCCACCGCTCGGGGAAACGCA
           ||||||||||||||||||||||||||||||||||||||||||||||||||||||||||||
153079301  GAGGAACCAGGAACGGCCAGTGAGAGGATGCACGACCTCGCCACCGCTCGGGGAAACGCA

153070790  AGGCACACAGCGATGAGGTGGTGTCTCTCCCCGTGGGATGGGCAAGCAGGCGAGGCTGGA
           ||||||||||||||||||||||||||||||||||||||||||||||||||||||||||||
153079241  AGGCACACAGCGATGAGGTGGTGTCTCTCCCCGTGGGATGGGCAAGCAGGCGAGGCTGGA

153070850  CAGGACCAAAGGCTGCTCGGGCTGTAGGAACCCAGGACCGCTCCCACGCTGCCAGTGCGA
           ||||||||||||||||||||||||||||||||||||||||||||||||||||||||||||
153079181  CAGGACCAAAGGCTGCTCGGGCTGTAGGAACCCAGGACCGCTCCCACGCTGCCAGTGCGA

153070910  GGGAACATGGGTTCAGCCACGCACCGGAGGTCACAGATGAGCAATGCTTGGTTGTGGCGT
           ||||||||||||||||||||||||||||||||||||||||||||||||||||||||||||
153079121  GGGAACATGGGTTCAGCCACGCACCGGAGGTCACAGATGAGCAATGCTTGGTTGTGGCGT

153070970  TGATAGGCACAGCCCAAGTCCCAGCAGAGCCACTGCCTGCTGTGCACCCCAGAGTCTTTC
           ||||||||||||||||||||||||||||||||||||||||||||||||||||||||||||
153079061  TGATAGGCACAGCCCAAGTCCCAGCAGAGCCACTGCCTGCTGTGCACCCCAGAGTCTTTC

153071030  TGGCTGACGGTGGTGGCTCAGACAGGCATGCGGGATGCCACTGGCAGCAGTGTCTGCAAC
           ||||||||||||||||||||||||||||||||||||||||||||||||||||||||||||
153079001  TGGCTGACGGTGGTGGCTCAGACAGGCATGCGGGATGCCACTGGCAGCAGTGTCTGCAAC

153071090  AGCCAAACAGTGGGAAGGACCGGAATGTCCATCCCCAGGGTCATAGACAAATACTCTGGG
           ||||||||||||||||||||||||||||||||||||||||||||||||||||||||||||
153078941  AGCCAAACAGTGGGAAGGACCGGAATGTCCATCCCCAGGGTCATAGACAAATACTCTGGG

153071150  GCTGTGATTCAGCAAGGAGCTTGGGCGAGCTGGACGCTTGGGGGAAACCTTGGCCAGCAA
           ||||||||||||||||||||||||||||||||||||||||||||||||||||||||||||
153078881  GCTGTGATTCAGCAAGGAGCTTGGGCGAGCTGGACGCTTGGGGGAAACCTTGGCCAGCAA

153071210  GTTGCCAAACAGGAGGTCAGGCAAGGAAAGAGAAGGGGATGAGGGAACGACACCCAGAGG
           ||||||||||||||||||||||||||||||||||||||||||||||||||||||||||||
153078821  GTTGCCAAACAGGAGGTCAGGCAAGGAAAGAGAAGGGGATGAGGGAACGACACCCAGAGG

153071270  TGTGACCCCGTGGGAACCTGTTTGCCACCTGCCTGGTGGAGATGCTGTTCACATGGCCCC
           ||||||||||||||||||||||||||||||||||||||||||||||||||||||||||||
153078761  TGTGACCCCGTGGGAACCTGTTTGCCACCTGCCTGGTGGAGATGCTGTTCACATGGCCCC

153071330  AGCCTCCCATGATGGGTCAGGTGCAGGGTGGTCAGATCCACGGGCGCAGAATCTCTGTTT
           ||||||||||||||||||||||||||||||||||||||||||||||||||||||||||||
153078701  AGCCTCCCATGATGGGTCAGGTGCAGGGTGGTCAGATCCACGGGCGCAGAATCTCTGTTT
153071390  TGAGCGGAGGCTGCTCTGCCCTGGCGCAGTGTCTAGCCCCGGCAACAGAGGACTGGGGAA
           ||||||||||||||||||||||||||||||||||||||||||||||||||||||||||||
153078641  TGAGCGGAGGCTGCTCTGCCCTGGCGCAGTGTCTAGCCCCGGCAACAGAGGACTGGGGAA

153071450  GGACCTATGGAAGGCTGACTCAGGCCCTGACAGACGGAGGTCTCCAGGGAGGTACGACAC
           ||||||||||||||||||||||||||||||||||||||||||||||||||||||||||||
153078581  GGACCTATGGAAGGCTGACTCAGGCCCTGACAGACGGAGGTCTCCAGGGAGGTACGACAC

153071510  CCATGCTGCCCTCAGGGCCAAGAGAGCAATGTCCCAGAGCCCATCAGGCTGGTGAGGGCA
           ||||||||||||||||||||||||||||||||||||||||||||||||||||||||||||
153078521  CCATGCTGCCCTCAGGGCCAAGAGAGCAATGTCCCAGAGCCCATCAGGCTGGTGAGGGCA

153071570  CAGGGGGCAGGGGACAAATTGAGTAGCCCACTCCGCGCGGACTACCTTTCCAGGCACACA
           ||||||||||||||||||||||||||||||||||||||||||||||||||||||||||||
153078461  CAGGGGGCAGGGGACAAATTGAGTAGCCCACTCCGCGCGGACTACCTTTCCAGGCACACA

153071630  CTCAGGCAGAGACCCAGACTCGGGACTTGCGGCTGCAAAACGGACGAGGCCCGGGCATAC
           ||||||||||||||||||||||||||||||||||||||||||||||||||||||||||||
153078401  CTCAGGCAGAGACCCAGACTCGGGACTTGCGGCTGCAAAACGGACGAGGCCCGGGCATAC

153071690  TTGGGTGCTGGGTCACCCTCCTAGGTGCTGGCTCCTGGCACCAGGGCAGCAAAGTGCTTC
           ||||||||||||||||||||||||||||||||||||||||||||||||||||||||||||
153078341  TTGGGTGCTGGGTCACCCTCCTAGGTGCTGGCTCCTGGCACCAGGGCAGCAAAGTGCTTC

153071750  GGCCGGAGGGCTGCGGGTCCCTGAAGCCGGATGGGCAGCGGGGAGATGGCAGACCCTCCG
           ||||||||||||||||||||||||||||||||||||||||||||||||||||||||||||
153078281  GGCCGGAGGGCTGCGGGTCCCTGAAGCCGGATGGGCAGCGGGGAGATGGCAGACCCTCCG

153071810  TAGGATGGTGTGCGTGAGCTGCGTGAGCGCCTGGGGCATCTCCTCCACGGAATGGCACCC
           ||||||||||||||||||||||||||||||||||||||||||||||||||||||||||||
153078221  TAGGATGGTGTGCGTGAGCTGCGTGAGCGCCTGGGGCATCTCCTCCACGGAATGGCACCC

153071870  GCACCAGGTGCCCCTGGCCTCGCCTGGCCTTGCCAGCCAGAGGACCAGTCCTGGACGGGG
           ||||||||||||||||||||||||||||||||||||||||||||||||||||||||||||
153078161  GCACCAGGTGCCCCTGGCCTCGCCTGGCCTTGCCAGCCAGAGGACCAGTCCTGGACGGGG

153071930  AGGGGGAGGACAAGAGCAGGTGCACAGGCCCCAGAACCCGGCTCCCTGACCCTGGCCAGA
           ||||||||||||||||||||||||||||||||||||||||||||||||||||||||||||
153078101  AGGGGGAGGACAAGAGCAGGTGCACAGGCCCCAGAACCCGGCTCCCTGACCCTGGCCAGA

153071990  ACGCAGAGCTGCCGGACGCACCTCAGACCACTCCCTGGCCCTCTGGTGGGCACGCCGGGG
           ||||||||||||||||||||||||||||||||||||||||||||||||||||||||||||
153078041  ACGCAGAGCTGCCGGACGCACCTCAGACCACTCCCTGGCCCTCTGGTGGGCACGCCGGGG

153072050  TGCACACCCGTCGCCCCGCTGGCGCCTGCCTCCGGGGCACGTGCGGGAGGGGCAGAGGCC
           ||||||||||||||||||||||||||||||||||||||||||||||||||||||||||||
153077981  TGCACACCCGTCGCCCCGCTGGCGCCTGCCTCCGGGGCACGTGCGGGAGGGGCAGAGGCC

153072110  TGGACCAGGAACCGCTGTGGTGAGAGCCAAAGGCGCTGGGACCTGCGGCCCACAGACCTT
           ||||||||||||||||||||||||||||||||||||||||||||||||||||||||||||
153077921  TGGACCAGGAACCGCTGTGGTGAGAGCCAAAGGCGCTGGGACCTGCGGCCCACAGACCTT

153072170  TGGACACCCCGGTGGGGGTGGGGGTGGGCTGGCGCCTGTGTTCCCGGGACCCGCGGCGCG
           ||||||||||||||||||||||||||||||||||||||||||||||||||||||||||||
153077861  TGGACACCCCGGTGGGGGTGGGGGTGGGCTGGCGCCTGTGTTCCCGGGACCCGCGGCGCG

153072230  AGGGGAGTGGGGGTGGGGCTAGGGGATAGGAGCAGGTGACGTCACCCGGGGTCCTGGGGC
           ||||||||||||||||||||||||||||||||||||||||||||||||||||||||||||
153077801  AGGGGAGTGGGGGTGGGGCTAGGGGATAGGAGCAGGTGACGTCACCCGGGGTCCTGGGGC

153072290  GCTCGGGAAGGCGGCGAGAGCCAGGCCACGTGACCCGCCCGCGGCAACGGCCCTGCGCTC
           ||||||||||||||||||||||||||||||||||||||||||||||||||||||||||||
153077741  GCTCGGGAAGGCGGCGAGAGCCAGGCCACGTGACCCGCCCGCGGCAACGGCCCTGCGCTC
153072350  TCCCGGGCCGGCGACGCGGAGGGGTCCGCGCTGCACCGCCGCCGCACCCCGGCCGCGTGA
           ||||||||||||||||||||||||||||||||||||||||||||||||||||||||||||
153077681  TCCCGGGCCGGCGACGCGGAGGGGTCCGCGCTGCACCGCCGCCGCACCCCGGCCGCGTGA

153072410  CCGCACTGCAGAGAGCGGCTGCGAGCAGGCGCCTGCGCACCGGCCCAAACGCCGAGCGAG
           ||||||||||||||||||||||||||||||||||||||||||||||||||||||||||||
153077621  CCGCACTGCAGAGAGCGGCTGCGAGCAGGCGCCTGCGCACCGGCCCAAACGCCGAGCGAG

153072470  CGAGGGAGAGGCACAGTCAGAGGGAACGCCCGCGCGGGGAGCCAGGGGCGCCCGACCCCG
           ||||||||||||||||||||||||||||||||||||||||||||||||||||||||||||
153077561  CGAGGGAGAGGCACAGTCAGAGGGAACGCCCGCGCGGGGAGCCAGGGGCGCCCGACCCCG

153072530  CCGCCGCCGCAGCGGCGCGCAGCCCCCGACGCGCCCTGTGGGGACCCGGACCAGGAGGGA
           ||||||||||||||||||||||||||||||||||||||||||||||||||||||||||||
153077501  CCGCCGCCGCAGCGGCGCGCAGCCCCCGACGCGCCCTGTGGGGACCCGGACCAGGAGGGA

153072590  CCCTGCCCCGGGAAAAGGTATCAGACCCACCCGGGGAGGGGGACGCTGGGTCCCGGGAGA
           ||||||||||||||||||||||||||||||||||||||||||||||||||||||||||||
153077441  CCCTGCCCCGGGAAAAGGTATCAGACCCACCCGGGGAGGGGGACGCTGGGTCCCGGGAGA

153072650  CTCCGCCGCCCCTCGCCTGTCCGCCCTAACAGAACTGAAGCAGCCTGCGGGGCAGCGGTC
           ||||||||||||||||||||||||||||||||||||||||||||||||||||||||||||
153077381  CTCCGCCGCCCCTCGCCTGTCCGCCCTAACAGAACTGAAGCAGCCTGCGGGGCAGCGGTC

153072710  CGGGGTCCGGGGTCCGGGGTCCGGGGTCCGGGGGCGGCGCGGGGATGAGGCCAAGACCCG
           ||||||||||||||||||||||||||||||||||||||||||||||||||||||||||||
153077321  CGGGGTCCGGGGTCCGGGGTCCGGGGTCCGGGGGCGGCGCGGGGATGAGGCCAAGACCCG

153072770  CAGGGACCACCCCCCCGGGGGGGCGGGGCGGGACAGGGGCGGGGCCTGGCTGCAGAGGCG
           ||||||||||||||||||||||||||||||||||||||||||||||||||||||||||||
153077261  CAGGGACCACCCCCCCGGGGGGGCGGGGCGGGACAGGGGCGGGGCCTGGCTGCAGAGGCG

153072830  CGGCCAGGGGAGGAGGGCGGCTGGGGGTGGGAGTGGACGTGGACGTGGACGTGGGCGTGG
           ||||||||||||||||||||||||||||||||||||||||||||||||||||||||||||
153077201  CGGCCAGGGGAGGAGGGCGGCTGGGGGTGGGAGTGGACGTGGACGTGGACGTGGGCGTGG

153072890  GGAGGGGTGGGCATTAACCTCGCTCTCGCCCGCTCGCATTCACAGGCTGTGGAGACCTGG
           ||||||||||||||||||||||||||||||||||||||||||||||||||||||||||||
153077141  GGAGGGGTGGGCATTAACCTCGCTCTCGCCCGCTCGCATTCACAGGCTGTGGAGACCTGG

153072950  GCTCCGACCCCAGTTCATCCCCCCACACCCCCGCCGCCCCGTGCCACCCTGGTCCGCGCT
           ||||||||||||||||||||||||||||||||||||||||||||||||||||||||||||
153077081  GCTCCGACCCCAGTTCATCCCCCCACACCCCCGCCGCCCCGTGCCACCCTGGTCCGCGCT

153073010  GGGAACCCTATCCTGCCCCTCGTGTCAGCCCGGCACTGGCCAGAATCGCGGGCATGGCGG
           ||||||||||||||||||||||||||||||||||||||||||||||||||||||||||||
153077021  GGGAACCCTATCCTGCCCCTCGTGTCAGCCCGGCACTGGCCAGAATCGCGGGCATGGCGG

153073070  TGACCATGCTGCAGGACTGGTGCCGGTGGATGGGGGTCAACGCTCGCAGGGGCCTGCTCA
           ||||||||||||||||||||||||||||||||||||||||||||||||||||||||||||
153076961  TGACCATGCTGCAGGACTGGTGCCGGTGGATGGGGGTCAACGCTCGCAGGGGCCTGCTCA

153073130  TCCTGGGCATCCCGGAGGACTGTGATGATGCCGAATTCCAAGAGTCCCTCGAGGCTGCCC
           ||||||||||||||||||||||||||||||||||||||||||||||||||||||||||||
153076901  TCCTGGGCATCCCGGAGGACTGTGATGATGCCGAATTCCAAGAGTCCCTCGAGGCTGCCC

153073190  TGAGGCCTATGGGACACTTTACAGTGCTAGGCAAAGCGTTTCGAGAGGAGGATAATGCCA
           ||||||||||||||||||||||||||||||||||||||||||||||||||||||||||||
153076841  TGAGGCCTATGGGACACTTTACAGTGCTAGGCAAAGCGTTTCGAGAGGAGGATAATGCCA

153073250  CCGCGGCCCTGGTCGAGCTCGACCGGGAAGTCAACTATGCTTTGGTCCCCAGGGAAATCC
           ||||||||||||||||||||||||||||||||||||||||||||||||||||||||||||
153076781  CCGCGGCCCTGGTCGAGCTCGACCGGGAAGTCAACTATGCTTTGGTCCCCAGGGAAATCC
153073310  CCGGCACTGGGGGCCCGTGGAACGTGGTCTTTGTGCCCCGTTGCTCAGGCGAGGAGTTTC
           ||||||||||||||||||||||||||||||||||||||||||||||||||||||||||||
153076721  CCGGCACTGGGGGCCCGTGGAACGTGGTCTTTGTGCCCCGTTGCTCAGGCGAGGAGTTTC

153073370  TCGGTCTCGGTCGCGTGTTCCACTTCCCGGAGCAAGAGGGGCAGATGGTGGAGAGCGTGG
           ||||||||||||||||||||||||||||||||||||||||||||||||||||||||||||
153076661  TCGGTCTCGGTCGCGTGTTCCACTTCCCGGAGCAAGAGGGGCAGATGGTGGAGAGCGTGG

153073430  CCGGCGCCCTGGGTGTGGGGCTGCGCAGGGTGTGCTGGCTGCGATCCATCGGTCAGGCGG
           ||||||||||||||||||||||||||||||||||||||||||||||||||||||||||||
153076601  CCGGCGCCCTGGGTGTGGGGCTGCGCAGGGTGTGCTGGCTGCGATCCATCGGTCAGGCGG

153073490  TCCAGCCCTGGGTGGAGGCCGTGAGGTGCCAGAGCCTGGGCGTGTTTTCCGGGAGGGACC
           |||||||||||||||||||||||||||||||||||||||| |||||||||||||||||||
153076541  TCCAGCCCTGGGTGGAGGCCGTGAGGTGCCAGAGCCTGGGTGTGTTTTCCGGGAGGGACC

153073550  AGCCAGCCCCAGGGGAGGAGTCCTTTGAGGTCTGGCTAGACCACACCACCGAAATGCTGC
           ||||||||||||||||||||||||||||||||||||||||||||||||||||||||||||
153076481  AGCCAGCCCCAGGGGAGGAGTCCTTTGAGGTCTGGCTAGACCACACCACCGAAATGCTGC

153073610  ATGTGTGGCAGGGGGTCTCGGAAAGGGAGAGGAGGAGGAGGCTGCTGGAAGGCTTGCGTG
           ||||||||||||||||||||||||||||||||||||||||||||||||||||||||||||
153076421  ATGTGTGGCAGGGGGTCTCGGAAAGGGAGAGGAGGAGGAGGCTGCTGGAAGGCTTGCGTG

153073670  GGACCGCCCTGCAGCTCGTGCACGCGCTCCTGGCGGAGAACCCCGCCAGGACGGCGCAGG
           ||||||||||||||||||||||||||||||||||||||||||||||||||||||||||||
153076361  GGACCGCCCTGCAGCTCGTGCACGCGCTCCTGGCGGAGAACCCCGCCAGGACGGCGCAGG

153073730  ACTGTCTGGCGGCCCTGGCCCAGGTGTTTGGAGACAACGAGTCCCAGGCGACCATCCGGG
           ||||||||||||||||||||||||||||||||||||||||||||||||||||||||||||
153076301  ACTGTCTGGCGGCCCTGGCCCAGGTGTTTGGAGACAACGAGTCCCAGGCGACCATCCGGG

153073790  TGAAGTGTCTGACCGCTCAGCAGCAGTCAGGCGAGCGTCTCTCAGCTTTCGTGTTGCGGC
           ||||||||||||||||||||||||||||||||||||||||||||||||||||||||||||
153076241  TGAAGTGTCTGACCGCTCAGCAGCAGTCAGGCGAGCGTCTCTCAGCTTTCGTGTTGCGGC

153073850  TGGAAGTGCTGCTGCAGAAGGCCATGGAGAAGGAGGCCCTGGCCAGAGCATCCGCCGACC
           ||||||||||||||||||||||||||||||||||||||||||||||||||||||||||||
153076181  TGGAAGTGCTGCTGCAGAAGGCCATGGAGAAGGAGGCCCTGGCCAGAGCATCCGCCGACC

153073910  GCGTGCGCCTGAGGCAGATGCTCACCAGGGCCCACCTTACTGAGCCTCTGGATGAAGCAC
           ||||||||||||||||||||||||||||||||||||||||||||||||||||||||||||
153076121  GCGTGCGCCTGAGGCAGATGCTCACCAGGGCCCACCTTACTGAGCCTCTGGATGAAGCAC

153073970  TGAGGAAGCTGAGAATGGCCGGGAGGTCTCCAAGTTTCTTGGAGATGCTGGGGCTCGTTC
           ||||||||||||||||||||||||||||||||||||||||||||||||||||||||||||
153076061  TGAGGAAGCTGAGAATGGCCGGGAGGTCTCCAAGTTTCTTGGAGATGCTGGGGCTCGTTC

153074030  GGGAGTCTGAGGCATGGGAGGCCAGTCTAGCCAGGAGCGTGAGAGCCCAGACACAGGAAG
           ||||||||||||||||||||||||||||||||||||||||||||||||||||||||||||
153076001  GGGAGTCTGAGGCATGGGAGGCCAGTCTAGCCAGGAGCGTGAGAGCCCAGACACAGGAAG

153074090  GGGCCGGTGCCCGGGCTGGTGCCCAGGCTGTTGCCAGAGCCAGCACTAAAGTAGAGGCGG
           ||||||||||||||||||||||||||||||||||||||||||||||||||||||||||||
153075941  GGGCCGGTGCCCGGGCTGGTGCCCAGGCTGTTGCCAGAGCCAGCACTAAAGTAGAGGCGG

153074150  TCCCAGGAGGTCCTGGTCGGGAGCCAGAGGGCCTCCTCCAGGCAGGAGGCCAGGAGGCTG
           ||||||||||||||||||||||||||||||||||||||||||||||||||||||||||||
153075881  TCCCAGGAGGTCCTGGTCGGGAGCCAGAGGGCCTCCTCCAGGCAGGAGGCCAGGAGGCTG

153074210  AGGAGCTCCTCCAGGAGGGGCTCAAGCCCGTCCTGGAGGAATGTGATAACTAGGTTGGGG
           ||||||||||||||||||||||||||||||||||||||||||||||||||||||||||||
153075821  AGGAGCTCCTCCAGGAGGGGCTCAAGCCCGTCCTGGAGGAATGTGATAACTAGGTTGGGG
153074270  CTGGGGAGGCAGCCCAGCGCGAGTCCTCCCCGGGCAAATAGGCTCCGAGGGCCCCGGGGC
           ||||||||||||||||||||||||||||||||||||||||||||||||||||||||||||
153075761  CTGGGGAGGCAGCCCAGCGCGAGTCCTCCCCGGGCAAATAGGCTCCGAGGGCCCCGGGGC

153074330  CTCCTCTCCTCCTCTCAGGCAGCAGGGCCCTGGAGACAGGCGGAGGCGGGGCCAGGGCCG
           ||||||||||||||||||||||||||||||||||||||||||||||| ||||||||||||
153075701  CTCCTCTCCTCCTCTCAGGCAGCAGGGCCCTGGAGACAGGCGGAGGCCGGGCCAGGGCCG

153074390  GTCCCTCACCCCACATCGGGATCGGGGCC  153074418
           |||||||||||||||||||||||||||||
153075641  GTCCCTCACCCCACATCGGGATCGGGGCC  153075613
```

**Supplementary Figure SF4.** **Analysis of the Inverted Repeat (IR) involved in Xq28 inversion** (NC_000023.11: g.(153065931_153075234)_(153075234_153084298)inv). **A.** Schematic of the Reference Structure involved in the Xq28 perfect inversion. IR arms are depicted as gray gross arrows; B indicates *Bcl*I restriction enzyme recognition sites. *PNMA6A*: PNMA family member 6A gene. NG_016372.2, NM_032882.6 → NP_116271.3 paraneoplastic antigen-like protein 6. *PNMA6B*: PNMA family member 6A pseudogene. **B.** 8.4kb-IR investigation by BLAST alignments: Centromeric Arm (8404 bp) *vs* Telomeric Arm (8428 bp). Gaps are highlighted in green (24 nucleotides: 8 + 15 +1); Mismatched nucleotides, in yellow (6 nucleotides). Location of IR internal limit coordinates allowed estimation of the actual range of the inversion: 1195bp. Xq28 inversion nickname: Xq28/1.2kb-inv. Nucleotide coordinates correspond to NC_000023.11.
