# Supplementary material for: Novel Strategy for Structural Variant Genotyping by Short‐Read Genomic Sequencing From Restriction‐Circles: Experimental and Bioinformatics Proof‐of‐Concept
Source: Hum Mutat. 2026 Oct 1;2026:2169350. doi: 10.1155/humu/2169350 (PMC13628565; doi:10.1155/humu/2169350)
Supplement: Supplementary file 2 [file HUMU-2026-2169350-s013.docx]

**Supplementary Text SX1: Detailed description of the bioinformatic pipeline.**

*Data preprocessing:*

Fastq file quality control was performed using FastQC (v.0.11.9). Reads were mapped to the human Reference Genome (GRCh38.p12) using the Burrows-Wheeler Aligner tool (BWA v.0.7.15), and sorting and indexing were performed using SAMtools (v.1.17). Subsequently, BAM files were transformed into Browser Extensible Data (BED) files using BEDtools (v.2.30) with the *bamtobed* command. The final BED file contained the read name, mapping characteristics, alignment orientation, Map Quality (MAPQ), and Concise Idiosyncratic Gapped Alignment Report (CIGAR) information (Figure 2, Supplementary Text SX2).

The BED file containing the coordinates and order number (5′–3′ sequential identification number) of each *Bcl*I restriction fragment from GRCh38.p12 (primary reference) was obtained using a Python script from HiC-Pro (Servant et al., 2015, doi: 10.1186/s13059-015-0831-x), with the *Bcl*I restriction enzyme (TGA^TCA) and GRCh38.p12 provided as inputs. An alternative reference was generated by removing the *Bcl*I recognition site plus or minus 2 bp from the BED file containing the *Bcl*I restriction fragments using an AWK command. This alternative reference was used to remove the *Bcl*I restriction sites from reads spanning it (Figure 2, Supplementary Text SX2).

*LPER definition:*

LPERs were identified, separated and characterized from all-PER dataset. *Bcl*I-site removal from reads and read mapping to the corresponding restriction fragment were performed using two sequential BEDtools *intersect* commands. First, the BED file containing all mapped reads was intersected with the alternative reference, generating a file containing read coordinate information in which the *Bcl*I restriction site had been removed. This output file was subsequently intersected with the restriction fragment BED file, and the corresponding restriction fragment order number was added to each read.

The final BED file contained all reads with the restriction recognition site removed and associated with the corresponding restriction fragment order number (Figure 2, Supplementary Text SX2). After p and q sides were assigned using AWK (Aho-Weinberger-Kernighan) scripting, LPER operator candidates were identified. The orientation of each read was evaluated and the distance from the 5′ end of each read to the closest *Bcl*I restriction site was calculated. Reads with a plus (+) orientation and distance values below 450 bp were assigned as “p”, whereas reads with a minus (−) orientation and distance values greater than −450 bp were assigned as “q”. The remaining reads were not classified as components of the LPER dataset and were therefore excluded from further analysis (Figure 1, Supplementary Text SX2).

LPER files were sorted and filtered, and only reads with non-duplicated names were retained. LPERs containing three or more reads with the same name were filtered out, ensuring that only read pairs for which both components had been assigned to either p or q (i.e., R1 and R2) were included. The information for each read was unified by read name and separated into two components, A and B, containing the R1 and R2 information, respectively. The final LPERs table contained non-duplicated information for each component (A and B), together with the assigned side (p or q) and the distance to the *Bcl*I restriction site (Figure 1, Figure 2, Supplementary Text SX2).

Finally, read quality scores were calculated using AWK. Reads were scored according to the length of the sections showing perfect matching (M). The M values of the corresponding CIGAR elements were summed for each read, and this value was divided by the maximum M CIGAR value (i.e., 151), resulting in a CIGAR score ranging from 0 to 1. In addition, MAPQ values, ranging from 0 to 60, were taken into account. The MAPQ value of each read was divided by the maximum MAPQ value (i.e., 60), resulting in a MAPQ score ranging from 0 to 1. The read quality scores were obtained by multiplying the CIGAR score by the MAPQ score. These values were compiled in the Main LPER Database (LPERs_24_human_Chr ref 45) (Supplementary Text SX2). The final TSV (tab-separated value) file contained all LPER information required to perform SV calling with our algorithm.

*Variant calling:*

The SV calling algorithm was based on LPER characteristics, including the order number of the restriction fragment, the restriction point side involved, and the counts associated with specific [A] and [B] (Figure 1 and Table 1).

To evaluate and complement the results obtained with the LPER-based SV calling approach, three conventional algorithms for detecting SVs from MPS datasets, Lumpy-SVtyper, SvABA, and Delly were applied. Because the *Bcl*I digestion followed by self-end fragment-ligation processes (circularization), required for LPER generation, may potentially confound conventional algorithms for SV genotyping, a BAM file containing only PERs and lacking LPERs was generated. For this purpose, the PER names corresponding to LPERs were filtered out from the original BAM file containing all mapped reads using SAMtools (Figure 2 and Supplementary Text SX2). The resulting filtered BAM file was used as input for the three programs with their recommended settings (Layer et al., 2014 doi: 10.1186/gb-2014-15-6-r84; Wala et al., 2018 doi: 10.1101/gr.221028.117; McKenna et al., 2010 doi: 10.1101/gr.107524.110).

For conventional SNV/indel analysis, the original BAM file containing all mapped reads was processed using the germline variant-calling algorithms recommended by GATK (GATK4 tools, ver. 4.1.4.0, URL: //gatk.broadinstitute.org/hc/en-us). HaplotypeCaller (DePristo et al., 2011 doi: 10.1038/ng.806) was used for variant calling, whereas the Variant Effect Predictor algorithm (VEP v.111) (McLaren et al., 2016 doi: 10.1186/s13059-016-0974-4) was used for VCF annotation (Figure 2).
