# Supplementary material for: Novel Strategy for Structural Variant Genotyping by Short‐Read Genomic Sequencing From Restriction‐Circles: Experimental and Bioinformatics Proof‐of‐Concept
Source: Hum Mutat. 2026 Oct 1;2026:2169350. doi: 10.1155/humu/2169350 (PMC13628565; doi:10.1155/humu/2169350)
Supplement: Supplementary file 3 [file HUMU-2026-2169350-s014.docx]

**Supplementary Text SX3: Calculation of the genomic coordinates of the DMD-causative SVs.**

Full characterization of the *DMD* genotype of the hemizygous patient with the DEL (*DMD* exons 45-54) and DUP (*DMD* exons 38-43) achieved by Luce et al (2021) using SNP-array and Sanger sequencing of the gap-PCR obtained products encompassing the breakpoints’ scars, at the cDNA level define the variants as NG_012232.1(NM_004006.2):c.6438+123812_8027+11362del and NG_012232.1(NM_004006.2):c.6291-5370_6291-5369ins[TAAAATGCAATTTCATTT;5326-5189_6291-5370], respectively. Assisted by Mutalyzer version 3.0.8 ([Mutalyzer 3](https://mutalyzer.nl/)) and [BLAST: Basic Local Alignment Search Tool](https://blast.ncbi.nlm.nih.gov/Blast.cgi), DEL and DUP are normalized at the genomic DNA level as NG_012232.1:g.1251506_1697982del and NG_012232.1:g.1122177_1122178ins[TAAAATGCAATTTCATTT; 990893_1122177], respectively. In order to compare with the genomic coordinates of the chromosome X GRCh38.p14, the output of our SV genotyping algorithm, the DMD exons 45-54 DEL may be written as NC_000023.11:g.31646629_32093105del, and the DMD exons 45-54 DUP, as NC_000023.11:g.32222433_32222434ins[AAATGAAATTGCATTTTA; 32222433_32353717], simplified as NC_000023.11:g.32222433_32353717dup.
